# Improvement of crop production in controlled environment agriculture through breeding

**DOI:** 10.3389/fpls.2024.1524601

**Published:** 2025-01-27

**Authors:** Krishna Bhattarai, Andrew B. Ogden, Sudeep Pandey, Germán V. Sandoya, Ainong Shi, Amol N. Nankar, Murukarthick Jayakodi, Heqiang Huo, Tao Jiang, Pasquale Tripodi, Chris Dardick

**Affiliations:** ^1^ Department of Horticultural Sciences, Texas A&M University, Texas A&M AgriLife Research and Extension Center, Dallas, TX, United States; ^2^ Department of Horticulture, University of Georgia, Griffin, GA, United States; ^3^ Horticultural Sciences Department, University of Florida, Everglades Research and Education Center, University of Florida – Institute for Food and Agriculture Sciences, Belle Glade, FL, United States; ^4^ Department of Horticulture, University of Arkansas, Fayetteville, AR, United States; ^5^ Department of Horticulture, University of Georgia, Tifton, GA, United States; ^6^ Department of Soil and Crop Sciences, Texas A&M University, Texas A&M AgriLife Research and Extension Center, Dallas, TX, United States; ^7^ Department of Environmental Horticulture, Mid-Florida Research and Education Center, University of Florida, IFAS, Apopka, FL, United States; ^8^ Council for Agricultural Research and Economics (CREA), Research Centre for Vegetable and Ornamental Crops, Pontecagnano-Faiano, SA, Italy; ^9^ United States Department of Agriculture-Agriculture Research Service (USDA-ARS), Appalachian Fruit Research Station, Kearneysville, WV, United States

**Keywords:** controlled environments, genetics, germplasm, genomics, high-throughput phenotyping, fruits and vegetables, breeding

## Abstract

Controlled environment agriculture (CEA) represents one of the fastest-growing sectors of horticulture. Production in controlled environments ranges from highly controlled indoor environments with 100% artificial lighting (vertical farms or plant factories) to high-tech greenhouses with or without supplemental lighting, to simpler greenhouses and high tunnels. Although food production occurs in the soil inside high tunnels, most CEA operations use various hydroponic systems to meet crop irrigation and fertility needs. The expansion of CEA offers promise as a tool for increasing food production in and near urban systems as these systems do not rely on arable agricultural land. In addition, CEA offers resilience to climate instability by growing inside protective structures. Products harvested from CEA systems tend to be of high quality, both internal and external, and are sought after by consumers. Currently, CEA producers rely on cultivars bred for production in open-field agriculture. Because of high energy and other production costs in CEA, only a limited number of food crops have proven themselves to be profitable to produce. One factor contributing to this situation may be a lack of optimized cultivars. Indoor growing operations offer opportunities for breeding cultivars that are ideal for these systems. To facilitate breeding these specialized cultivars, a wide range of tools are available for plant breeders to help speed this process and increase its efficiency. This review aims to cover breeding opportunities and needs for a wide range of horticultural crops either already being produced in CEA systems or with potential for CEA production. It also reviews many of the tools available to breeders including genomics-informed breeding, marker-assisted selection, precision breeding, high-throughput phenotyping, and potential sources of germplasm suitable for CEA breeding. The availability of published genomes and trait-linked molecular markers should enable rapid progress in the breeding of CEA-specific food crops that will help drive the growth of this industry.

## 1 Introduction

Increased food production with higher nutritional content is required to feed the growing global human population, particularly in urban centers. Furthermore, the challenge to sustainably increase food and nutrition is exacerbated in the face of dynamic environmental and biotic threats because of a rapidly changing climate. Controlled environment agriculture (CEA) is a climate-resilient system that offers promise toward food security and production sustainability (Specht et al., 2014; O’Sullivan et al., 2019; Walsh et al., 2022). CEA has revolutionized horticultural production by enabling year-round cultivation, protection from adverse weather conditions, pests, and diseases, and precise control over environmental factors such as temperature, humidity, light, and CO_2_ levels (Shamshiri et al., 2018). Such control allows growers to optimize crop growth, quality, and yield while minimizing resource use (Kozai, 2013; Cowan et al., 2022; Gargaro et al., 2023). Rising as an alternative crop production system, CEA offers the potential to increase production per unit area and quality due to enhanced control of growing conditions (Nie and Zepeda, 2011; Coyle and Ellison, 2017; Ares et al., 2021). CEA comprises a wide array of controls in production facilities ranging from basic, such as plastic tunnels, advanced, such as greenhouses, to complex, such as vertical indoor farms (Mitchell, 2022). While some CE facilities like polytunnels and greenhouses have been around since the 19th century, technological innovations like indoor vertical farms are new additions and are continuously being optimized.

In the US, CEA production is currently represented by tomatoes (59%), fresh herbs (12%), cucumbers (7%), lettuce (6%), peppers (3%), strawberries (1%), and other unspecified crops (12%) (Dohlman et al., 2024). Initially, the focus of CEA farms was leafy greens and herbs because of their fitness and short production cycles. However, to serve a balanced diet, production of a diverse set of crops is needed. In some European and Asian countries, fruits and vegetables like leafy greens, melons, peppers, strawberries, tomatoes, and cucumbers are largely grown in CEs, and in the Americas, the production of cane and bramble fruits is expanding under high tunnels (Demchak, 2009; Cowan et al., 2022; Ayinde et al., 2024). The number of CEA operations doubled and reached 3,000 between 2009 to 2019 and production increased by 56% to 786 million pounds (Dohlman et al., 2024). In 2014, CEA production contributed $769 million to the US economy. However, increased competition from imports decreased the revenue to $626 million in 2019 (Dohlman et al., 2024).

## 2 Current challenges and opportunities

High capital and operational costs, high energy requirements, and limited crop diversity are the bottlenecks in the rapid expansion of CE production (Cowan et al., 2022; Dsouza et al., 2023). CEA facilities require significant capital investment, often taking 5–7 years to become profitable (Agrilyst, 2017). The startup costs of a vertical farm can range from $150 to $400 per 0.093 m^2^ (1 foot^2^) as compared to $50 to $150 for a greenhouse (Stein, 2021). The application of electronic sensors, mechanization, and robotic systems further incurs higher costs. Although higher costs could be compensated by increased productivity, yield, and high-quality produce fetching higher premiums, reducing energy consumption and incurred costs remain as major challenges.

The rapidly evolving production technologies, specialized inputs, and targeted consumer markets present opportunities to diversify crop production and maximize production efficiency relieving financial burden. The high startup costs are largely due to infrastructure, labor, and the energy needed for climate control and lighting. However, the development and adoption of supplementary technologies such as light-emitting diodes (LEDs), solar panels, and other advancements are gradually helping to reduce these costs (Teitel et al., 2012; Mohareb et al., 2017; van Iersel, 2017). With increasing investment, ongoing research is focused on enhancing crop yields, lowering operational expenses, and optimizing LED lighting for prolonged production, efficient nutrient uptake, and improved production platforms (Touliatos et al., 2016).

Despite research and technological advancements in crop production, challenges related to environmental impact, supply chain, and consumer interest prevail. The establishment of CEA facilities around communities increases awareness and improves food access (Sheng, 2018; Beacham et al., 2019; Stein, 2021), leading to increased consumer preference for CEA-produced crops (Ares et al., 2021). These facilities may reduce the transportation costs and carbon footprint associated with the supply chain (Sheng, 2018; Stein, 2021). Despite these benefits, CEA production is currently limited to leafy greens, tomatoes, cucumbers, and some berries. With technological advances and public–private interest, a wider range of crops can be anticipated. One of the important factors in crop diversification is the availability of CEA-optimized plant materials. Most of the currently produced cultivars have been bred for field-based agriculture. Cultivars developed for open fields may not account for the enclosed, limited space and lighting requirements observed in CEA. Therefore, there is a need to breed cultivars with unique crop characteristics that help plants thrive in these facilities. This review will discuss breeding strategies and programs on different crops suitable for CEA. We will first introduce the main breeding targets to discuss the progress made in commonly grown crops. We then debate the importance of next-generation sequencing technologies, precision breeding, and advanced phenotyping technologies and their use in breeding and present the prospects of diversifying the crops in CEA.

## 3 Breeding for CEA

**Figure 1:**
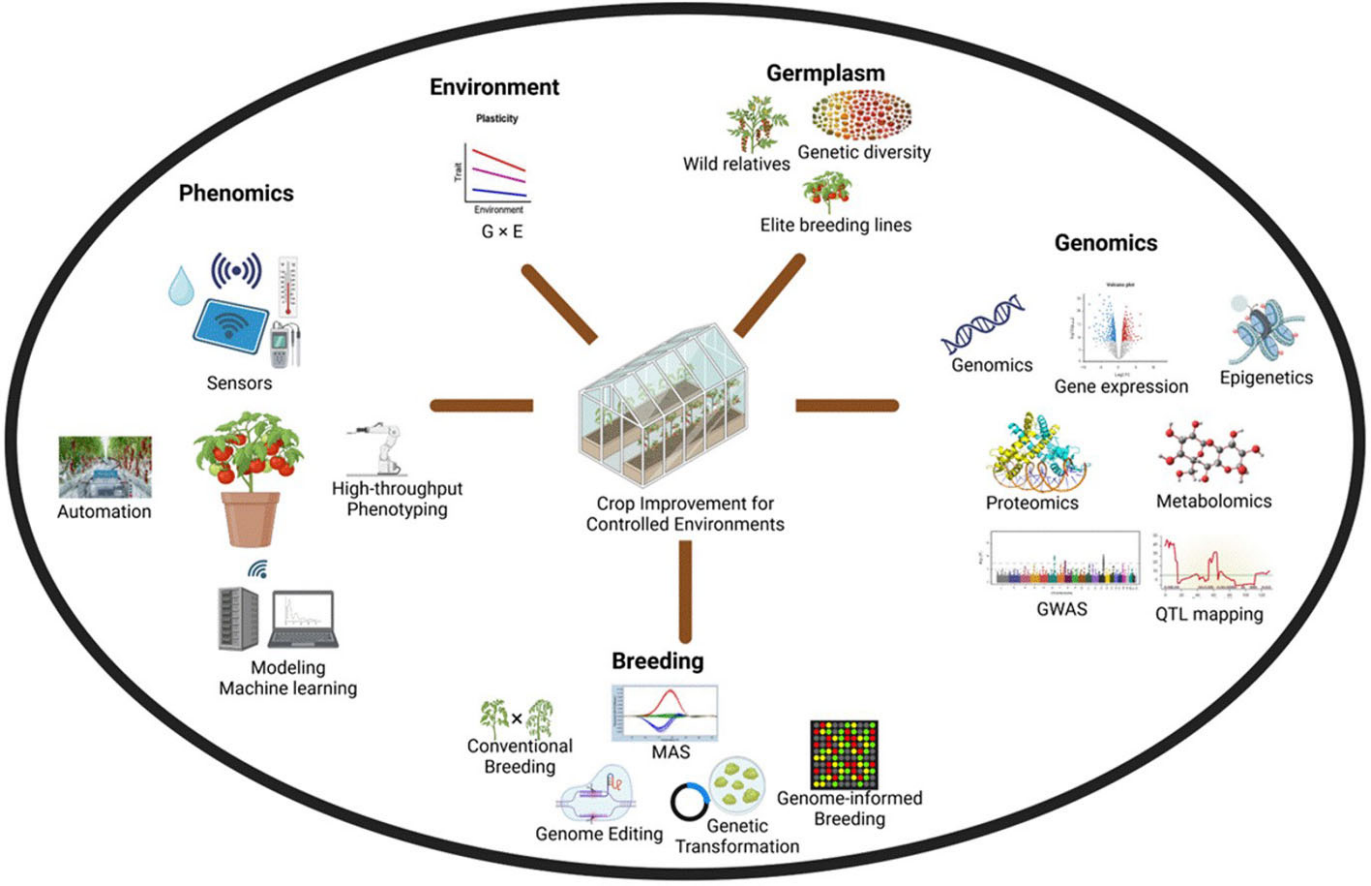
Leveraging multidisciplinary technologies to improve crop cultivars for controlled environment agriculture.

**Figure 2:**
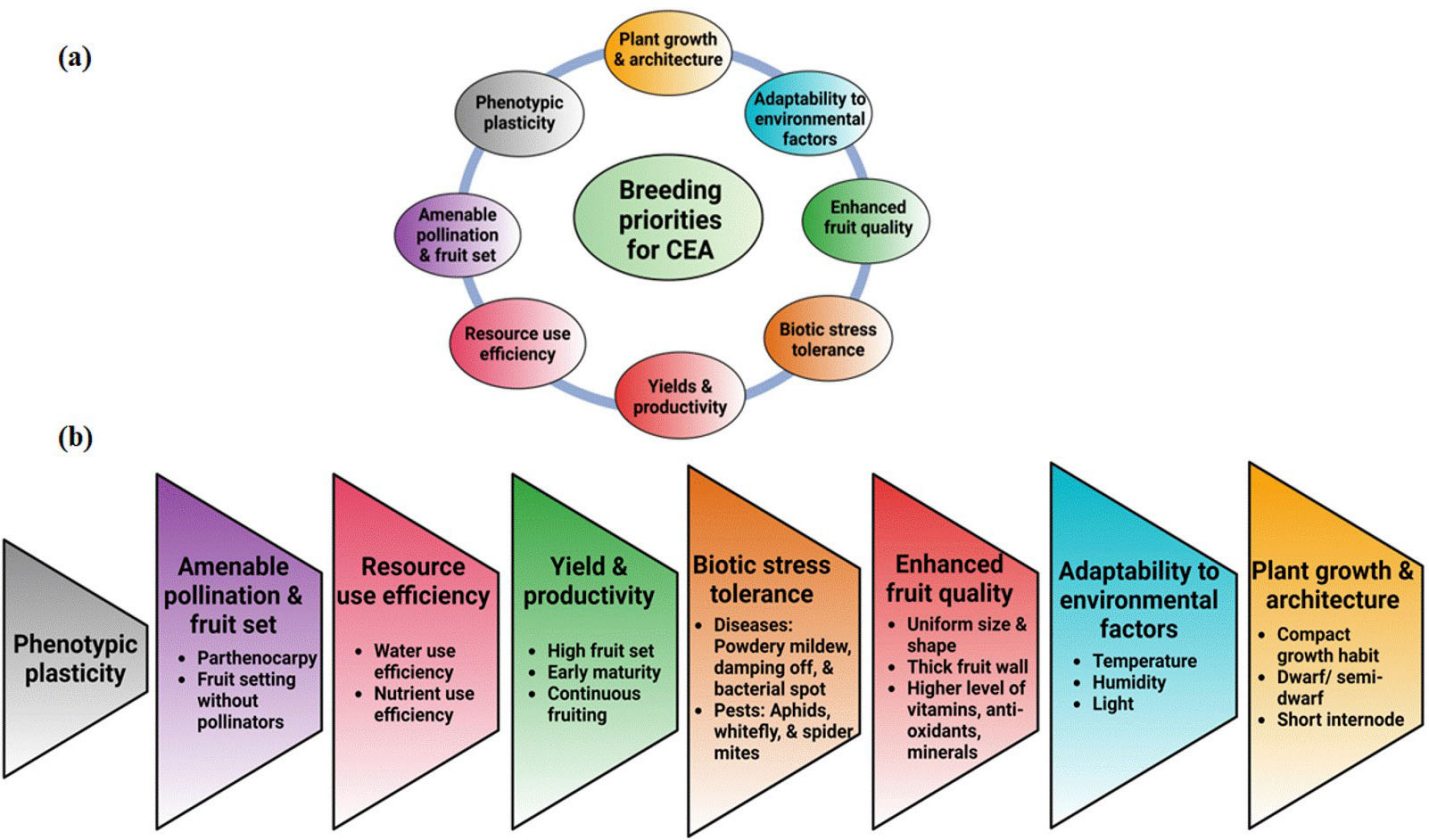
**(A)** Breeding priorities for CEA. **(B)** Targeted traits belonging to specific breeding priorities. This is a representative trait list, which may vary from program to program.

Breeding for CEA can make a significant impact on food production as crop and cultivar choices drive the profitability of the farms. Crop adaptability and performance in CEA can differ significantly from field conditions (Gruda, 2005). Crop improvement for CEA is a novel field with unique challenges and requires the application of multidisciplinary approaches (**Figure 1**). Uniform optimal conditions for plant growth and development necessitate the development of cultivars tailored to CEA. A concept of one promising cultivar that could be potentially grown globally should attract the interest of seed companies. Optimal growing conditions and enhanced control should enable plant breeders to refocus on quality traits like taste, nutrition, and health benefits (Kreuger et al., 2018). In addition, CEA has been leveraged for speed breeding and support cultivar development in cereals (Alahmad et al., 2018), legumes (Peck et al., 2023), and vegetables (Gimeno-Páez et al., 2024), potentially becoming an integrated part of all breeding programs in the future. CEA benefits from multidisciplinary technology and breeding efforts are required not only to improve agronomic traits but also traits that allow applications of newer technology such as automation to evolve as an efficient and sustainable production system (**Figure 2**).

**Figure 3:**
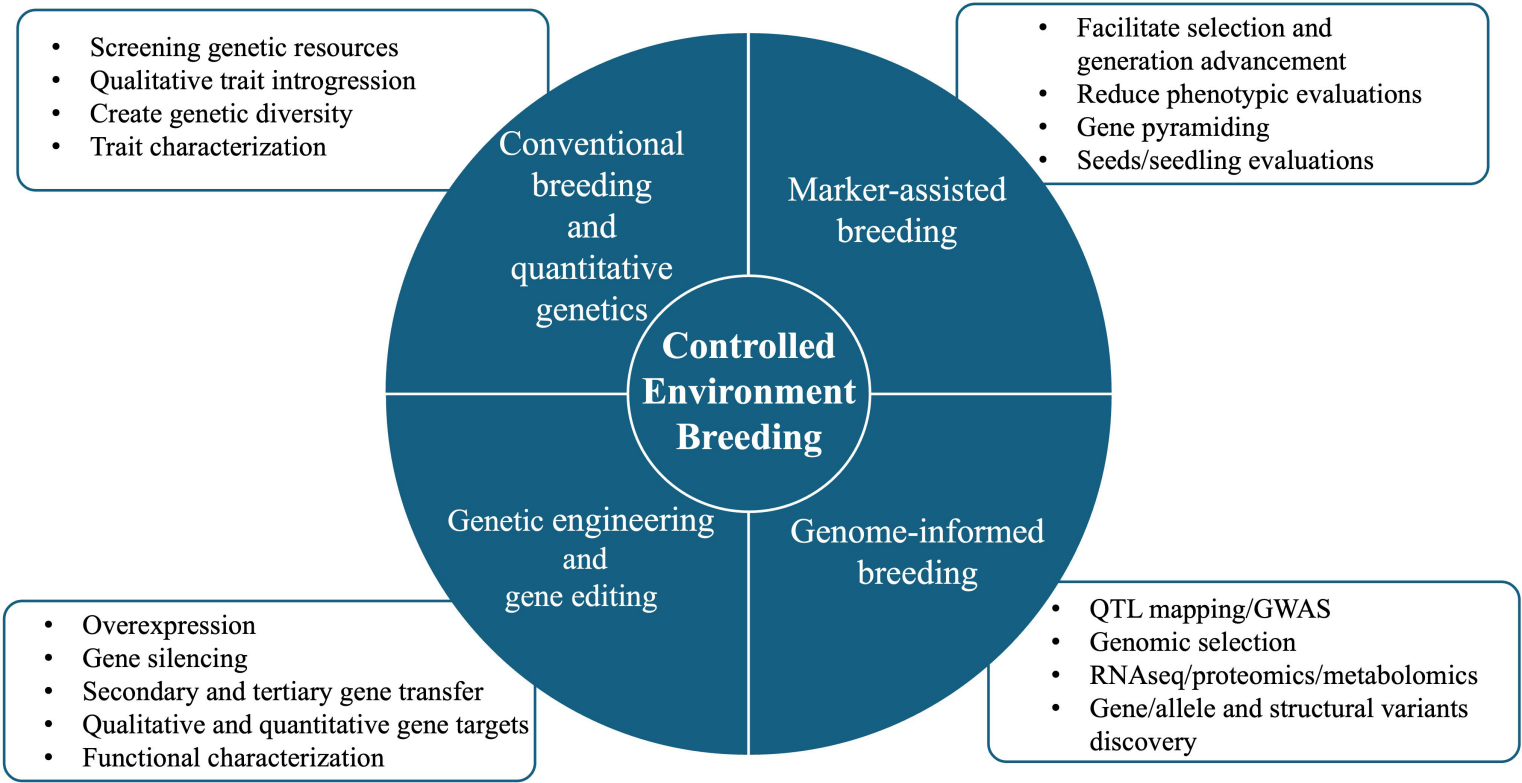
Strategies, techniques and their applications in breeding crops for controlled environments.

The goal of breeding is to predict and increase genetic gain over generations (time) to the available genetic variation, intensity, and accuracy of the selection for human, economic, and environmental benefit. The high initial investment and operating costs necessitate the development of highly productive and resource-efficient crops to ensure economic viability (Kozai, 2013). CEA has elucidated the need for genetic gains in traits that increase crop fitness driven by the growing conditions and apply to multiple crops. Because of variations in the CEA production system, trait importance in these systems differs accordingly. While not critical for single-tier systems like greenhouses and high tunnels, crop canopy is pivotal as small stature is sought to maximize space utilization in multi-tier production systems like indoor and vertical farms. In leafy greens, smaller canopies allow high-density planting and increase fitness in multi-tier systems. Similarly, shorter crop cycles maximize productivity, allowing multiple crop cycles in each space. CEA growers can produce 11–12 cycles of lettuce, as compared to 1–2 crop cycles grown per year in open fields (USEPA, 2007). The higher number of crop cycles helps to offset the production costs. One of the limitations of CEA is its high energy usage for temperature regulation and lighting. With high-density planting, meeting photosynthetic photon flux density parameters requires high-intensity lights. Therefore, breeding cultivars that perform well in low light can reduce energy usage, especially in the CEA systems relying on supplemental or complete electric lighting. Breeding for enhanced cold or heat tolerance could minimize energy use in cooler regions like northern Europe or warmer regions like the Southern US or the Middle East across all CEA production systems. Crop production in indoor and vertical farms relies on soilless systems, such as hydroponics to increase nutrient absorption, improve sanitation, and minimize diseases and pests. It is essential to breed plants that grow well in such systems to meet industry needs. Other traits for consideration in CEA breeding include adaptation to supplemental CO_2_, enhanced nutritional content, improved flavor, pollination efficiency, parthenocarpy, and resistance to prominent CE diseases and pests. A comprehensive depiction of breeding strategies for CEA is shown in **Figure 3**.

Crop germplasm improved through years of field breeding can be harnessed to expedite cultivar development for CEA for traits like disease resistance, yield, and heat tolerance. Changes in breeding priorities in CEA require germplasm development for unique traits like adaptation to limited space, low light intensity, and fruit set. While a vigorous and deep root system is desired for lettuce in field production to develop crop resilience, readily available water and nutrients in CEA will benefit from the germplasm that allows flexibility to select for high-shoot biomass while maintaining an optimum root system. Similarly, year-round production in CEA will need germplasm to breed for dwarf tomato cultivars with shorter crop cycles. In addition, germplasm development for traits like fruit quality, novel ideotypes, light types and intensity, and soilless substrate culture are some of the traits that will need specialized germplasm development for CEA.

## 4 Major crops and their breeding targets for CEA

### 4.1 Lettuce

Lettuce (*Lactuca sativa* L.) is among the topmost cultivated crops in CEA for food production in the US (USDA-NASS, 2019). Lettuce has seven morphological types, namely, crisphead subdivided into Batavia and iceberg, butterhead including Boston and bibb, romaine or cos lettuce, Latin, and leaf or cutting lettuce, which are most common in field production worldwide (Ryder, 1999); however, in the US, the most popular types are crisphead, romaine, and leaf lettuce (USDA-NASS, 2022). More ancient types of lettuce include stem lettuce also known as “stalk” or “asparagus” lettuce and the oil seed type (Hayes, 2018). Traditionally, the crop has been improved for field adaptation (Hayes, 2018). While romaine and icebergs have been historically improved through breeding for head weight and morphology for field production, the most cultivated types in CEA are romaine, butterhead, and leaf lettuce and their subtypes. CE growers may require cultivars that offer novel morphological types than those grown in fields. A combination of leaf and crisphead lettuce commonly known as “crunchleaf” or “summercrisp” is currently desired by the industry.

Several key traits important for field production including high water- and nutrient-use efficiency (WUE and NUE) and resistance to biotic and abiotic stresses are also important in CEA (Sandoya, 2019). The CEA industry will benefit from lettuce cultivars improved for unique traits including nutritional value, new leaf shapes and colors, and the ability to adapt to new horticultural technologies including light use efficiency and mechanical harvesting. Variations in phylloquinone, tocopherols (alpha and gamma), and ascorbic acid (vitamin C) content in hydroponically grown lettuce demonstrate the opportunity to enhance metabolites beneficial for human health (Murray et al., 2023). Similarly, genetic variability within *L. sativa* for morphological traits like leaf shape, color, and texture can be utilized to develop cultivars for niche markets (Sandoya, 2019). Opportunities exist to create newer shapes and color variations from green to red that could be beneficial to CEA producers.

Major concerns for lettuce producers are diseases caused by plant pathogens. Most of the genetic studies in *L. sativa* and related species have been conducted for disease resistance. There is extensive germplasm testing either in a greenhouse (or lab-associated assay) or on the field for diseases such as downy mildew (DM) caused by *Bremia lactucae*. There are at least 51 genes and 15 QTLs controlling DM resistance (Parra et al., 2016). However, the fungus rapidly evolved overcoming host resistance into different races, of which 10 races are present in the US and 23 races are in the European Union (IBEB, 2024) while the *B. lactucae* race structure in other lettuce-producing regions of the world is unknown (Wu et al., 2018). The resistance to DM is widespread across several chromosomes in the lettuce genome and multiple germplasms of *L. sativa* and wild types of *L. serriola* and *L. saligna* (see Parra et al., 2016 for details). Further resistant loci were relatively recently identified in *L. sativa* (Parra et al., 2016; Simko et al., 2021). Other diseases including Fusarium wilt, Verticillium wilt, Sclerotinia drop, and Corky root rot are a concern to field producers due to their soil-borne nature (Raid and Sandoya-Miranda, 2024). It is expected that the pathogens causing these diseases, *Fusarium oxysporum* f. sp. *lactucae*, *Verticillium dahliae*, and *Rizhorhapis suberifaciens*, would be less problematic when the crop is produced hydroponically or in soilless substrates. However, a soilborne disease, Pythium wilt (Davis, 2018), has been reported on lettuce cultivated in CEA (Tsoukas et al., 2023). The disease has become problematic in fields and the pathogen has been identified and reclassified as *Globisporangium uncilunatum* (syn *P. uncinulatum*) (Slinski et al., 2024). Several of these soilborne pathogens are believed to be seed-borne. Further investigation on the survival of these pathogens from contaminated seeds on specific media (water, growing media including rockwool, vermiculite, etc.) can reveal the potential outbreaks of these pathogens.

While powdery mildew (PM) caused by *Golonovinomyces cichoracearum* is not a significant problem in the field except during conducive conditions, the disease is more problematic in greenhouses (Simko et al., 2014). The intent of year-round production in CEA creates the need for improved germplasm with resistance. There are few resistance sources for PM in the wild and cultivated lettuce, especially in butterhead lettuce (Lebeda, 1985, 1994; Lebeda et al., 2014; Simko et al., 2014). Resistance to other diseases concerning the CEA lettuce industry needs further investigation. There are no known sources of resistance against *P. uncilunatum* or any *Pythium* spp. and *C. latucae-sativae* (Raid, 2018). It remains unknown if *F. oxysporum* f. sp. *lactucae, V. dahlia*, or *R. suberifaciens* will cause disease outbreaks on lettuce in soilless media.

Lettuce is best adapted to temperatures below 28°C during the day and 23°C at night (Hayes, 2018). Higher temperatures could lead to early bolting and tipburn. Similarly, the crop suffers damage when exposed to longer periods of cold temperatures below 18°C during the day and 10°C, at night, respectively (Hayes, 2018). The rising temperatures across the globe are affecting both marketability and promoting physiological disorders (Lafta et al., 2017, 2021; Raid and Sandoya-Miranda, 2024). The need for cooling or heating of CEA operations results in increased production costs. The development of cold- and heat-tolerant cultivars is desired for both field and CEA production. Genetic variability has been identified within *L. sativa* for heat tolerance in romaine, butterhead, crisphead, and leaf lettuce (Lafta et al., 2017, 2021; Kreutz et al., 2021; Raid and Sandoya-Miranda, 2024). However, the genetics underlying the trait are yet to be identified. Further efforts are underway to identify germplasm that tolerate warmer temperatures in greenhouses to improve heat tolerance. Selection against bolting and tipburn is beneficial to both field and CEA lettuce production. While genetic variation is present for bolting (Rosental et al., 2021), tipburn, a physiological disorder, is partially controlled by a genetic component (Hayes, 2006; Macias-González et al., 2019) and needs further research.

For CEA, lettuce should be efficient in several factors including nutrient uptake and utilization. Lettuce cultivars with high WUE have been identified (Eriksen et al., 2016; Macias-González et al., 2021). Nitrogen and phosphorus uptake are known to be genetically controlled in lettuce (Macias-González et al., 2021; Kreutz et al., 2022). The genetics of WUE and nitrogen uptake are complex involving multiple loci distributed across the genome (Macias-González et al., 2021). It is important to note that these genetics are described for field lettuce and only phosphorus uptake has been investigated in hydroponic settings (Kreutz et al., 2023). Salinity is posing a concern in areas that are aquifer-dependent or close to saltwater. Salt intrusion into aquifers has affected the crop by higher salt levels in soils (Miceli et al., 2003). Lettuce cultivars tolerant to salinity could alleviate this problem (Xu and Mou, 2015; Eriksen et al., 2016) and could be used for saltwater-based aquaponics systems in CEA.

Most genetic studies in lettuce have been conducted using biparental mapping populations. There are few publicly available molecular markers for use in marker-assisted selection (MAS) specifically for disease resistance (Michelmore, 2018). These markers are limited to single locus inherited traits. Limited genome-wide association studies (GWAS), including resistance for bacterial leaf spot, DM, and shelf life, have been conducted using diverse germplasm sets (Sthapit Kandel et al., 2020; Kandel et al., 2022; Simko et al., 2022). As more phenotypic traits are mapped using GWAS populations, genomic selection (GS) will be employed to breed lettuce for polygenic traits.

### 4.2 Spinach

Spinach (*Spinacia oleracea* L.) is widely recognized for its exceptional nutritional profile. It is packed with essential vitamins (A, C, K, and folate), minerals (iron and calcium), antioxidants (lutein, zeaxanthin, and flavonoids), and dietary fiber (Drewnowski and Gomez-Carneros, 2000; USDA FoodData Central, 2024). Traditionally, spinach is grown in open fields in the US, predominantly in California (April to October) and Arizona (November to March). Growing consumer demand for nutritious foods, particularly leafy greens like spinach, has spurred advancements in cultivation methods. There is a growing shift towards year-round production in CEA to meet the demand while minimizing environmental impact (Garcia et al., 2023). Breeding spinach specifically for CEA has become vital to meet the growing shift. Key breeding objectives include optimizing yield, maintaining or enhancing the nutritional profile under controlled conditions, and developing varieties that meet consumer demands for health-promoting vegetables.

#### 4.2.1 Growth and yield optimization

Maximizing growth rates is essential for improving turnover and productivity. Optimized light conditions are crucial for enhancing growth rates (Folta, 2019). Genetic studies have identified loci associated with growth rate, presenting potential targets for breeding programs (Joshi et al., 2022). Additionally, factors such as irradiance, nutrient solution temperature, and nutrient levels significantly affect spinach growth rates (Gent, 2017). Improving nitrogen use efficiency through genetic means could further enhance growth (Chan-Navarrete et al., 2016). Breeding efforts should focus on increasing yield potential by selecting traits such as increased leaf area, higher leaf-to-stem ratio, and reduced bolting.

#### 4.2.2 Nutritional quality

Spinach shows variations in its nutritional composition across different accessions and cultivars. Studies have reported a wide range of concentrations for key nutrients such as nitrate (0.21–3.83 mg/g FW), oxalate (2.38–34.72 mg/g FW), vitamin C (ascorbic acid) (0.51–1.30 mg/g FW), and carotenoids (0.18–0.58 mg/g FW) (Wang et al., 2018a). Organic cultivation typically results in higher levels of ascorbic acid and flavonoids and lower nitrate content compared to conventional methods (Koh et al., 2012). Light intensity also impacts nutrient composition, with low light leading to decreased ascorbate and increased oxalate and nitrate levels (Proietti et al., 2004). Despite these insights, the genetic basis of vitamin C content is still poorly understood, with limited research utilizing association mapping (Rueda et al., 2021). Further genetic studies and breeding efforts are needed to elucidate the mechanisms behind ascorbic acid, mineral, and phytonutrient content, ultimately leading to varieties with enhanced nutritional value (Kim et al., 2018). Additionally, breeding for high levels of essential minerals like iron, magnesium, and calcium is crucial, and genetic markers associated with these mineral contents have been identified (Qin et al., 2017). Breeding programs aim to develop spinach cultivars with improved nutritional profiles by increasing vitamin C and carotenoids while reducing nitrate and oxalate accumulation (Wang et al., 2018a; Rashid et al., 2022). Research indicates that nitrate content varies significantly depending on genotype, environmental conditions, and fertilizer use (Abubaker et al., 2005; Wang et al., 2018a; Luetic et al., 2023). Therefore, selecting against nitrate accumulation is important to ensure safer consumption.

#### 4.2.3 Leaf quality and consumer acceptance

Attributes such as dark green color, tender texture, and mild flavor are critical for consumer acceptance (Batziakas et al., 2019). Softer, more palatable leaves are essential for fresh consumption (Batziakas et al., 2019; Liu et al., 2021). Genetic mapping has identified markers linked to desirable leaf textural traits (Xu et al., 2017b; Liu et al., 2021). Furthermore, specific loci associated with leaf length, width, and petiole length important for determining leaf texture and tenderness have been identified and used in breeding (Liu et al., 2021).

#### 4.2.4 Pest and disease resistance

Although CEs typically reduce the risk of pest infestations, diseases such as damping-off, caused by *Pythium* species, and fungal infections pose challenges in CEA, prompting breeding programs to emphasize genetic screening for disease resistance (Syu et al., 2024). Genetic host resistance is effective against common greenhouse diseases and pests (Kozai, 2013). Resistance to fungal pathogens, such as DM and leaf spot, is vital for maintaining high crop survival and yield. GWAS has identified single-nucleotide polymorphism (SNP) markers linked to resistance against significant pests and diseases (Bhattarai et al., 2021a). This includes resistance to Verticillium wilt caused by *V. dahliae* (Shi et al., 2016b), leaf spot caused by *Stemphylium vesicarium* and *S. beticola* (Shi et al., 2016a), anthracnose caused by *Colletotrichum spinaciae* (Awika et al., 2020), and white rust (Awika et al., 2019; Shi et al., 2022). Research on genetic bases of DM resistance has added valuable insights for resistant cultivar development (Bhattarai et al., 2020, 2021b, 2022b, 2022a, 2023).

#### 4.2.5 Harvest and post-harvest qualities

Harvest and post-harvest qualities are critical for spinach cultivated in CEA, focusing on maintaining freshness, nutritional quality, and ease of harvesting. Genetic research has identified specific genes associated with enhanced post-harvest longevity in spinach, crucial for breeding varieties that remain fresh and nutritionally valuable over extended periods (Xu et al., 2017b). Traits such as easy leaf detachment and regrowth capabilities are vital for improving harvesting efficiency in CEA systems (Al-Kodmany, 2018; Beacham et al., 2019). Genetic markers linked to these traits enable breeders to develop spinach varieties that streamline harvesting processes enhancing crop management and productivity (Hirakawa et al., 2021).

#### 4.2.6 Genomics and breeding advances

Recent advances in genomics, including genome assemblies, GWAS, and MAS, have revolutionized gene and quantitative trait locus (QTL) identification, trait selection, and cultivar development. These advancements have significantly improved the efficiency and precision of spinach breeding for CEA (Bhattarai et al., 2021a). Genome assemblies facilitate GWAS for traits such as disease resistance, bolting, and leaf morphology (Cai et al., 2021; Hirakawa et al., 2021; Bhattarai et al., 2022a, 2022b). MAS and GS methods further enhance breeding efficiency, particularly for DM resistance (Bhattarai et al., 2021a, 2022b, 2022a; Simko et al., 2021; Joshi et al., 2022; Shi et al., 2022). Genomic analyses also provide insights into spinach domestication, population structure, and sex chromosome evolution (Cai et al., 2021; Ma et al., 2022b). Resources like SpinachBase support spinach genomics research by offering genomic data and analytical tools (Collins et al., 2019). GWAS studies have analyzed spinach accessions to identify loci associated with nutritional elements and leaf traits, including texture (Ji et al., 2024). These studies provide valuable insights for breeding programs aiming to enhance leaf tenderness. The deep green color of spinach, indicative of its high nutritional value, is also a focus of genetic research (Sokolova and Solovyova, 2023). Some studies by Cai et al. (2018) have advanced the understanding of pathways regulating chlorophyll production in spinach. Targeting these genetic pathways could help develop cultivars with enhanced coloration.

These technologies have enabled the identification and incorporation of desirable traits, such as leaf texture, color, and flavor, into breeding programs, ensuring new varieties meet nutritional, agronomic, and market demands. Furthermore, genomic and molecular approaches show promise in addressing production challenges, such as biotic and abiotic stresses, and in providing improved breeding materials and strategies (Bhattarai et al., 2021a). Continued advancements in functional genomics will enhance our understanding of complex traits in spinach and accelerate the development of improved cultivars (Cai et al., 2021; Das et al., 2023; Tawfik, 2023; Ji et al., 2024). Integrating genomic insights and advanced breeding techniques is essential for developing varieties optimized for CEA systems. Continued research and innovation in this field will support sustainable spinach production, meet the growing demand for health-promoting vegetables, and address the challenges of modern agriculture.

### 4.3 Tomato

Tomato breeding has specialized based on the market, product type, and cultural requirements such as fresh market, processing, CEs, or home garden (McKenzie, 2014). For varietal development, unique breeding strategies are needed for specific growing conditions and market challenges. Breeding tomatoes for CEs is evolving to meet the demands of production facilities such as indoor farms, vertical farms, greenhouses, and their modified forms. Most single-tier CE farms currently grow indeterminate tomatoes that can grow for an extended period and determinate large-fruited tomatoes, with or without grafting. Introducing tomatoes to vertical farms is yet to be realized due to a lack of optimized cultivars. Kozai (2013) recommended plant height to be approximately 30 cm to optimize space and ensure multi-tier production in platforms established for leafy vegetables. Modifications in plant architecture, morphology, and size are implemented to develop dwarf plants that fit in CEA. Various genes—*DWARF (D)*, *SELF-PRUNING (sp)*, *miniature (mnt)*, and *Dumpy (dpy)*—contribute to reducing plant stature (Bishop et al., 1996; Pnueli et al., 1998; Koka et al., 2000; Martí et al., 2006). Moreover, meristem traits such as side shoots and branching need optimization. Canopy reduction results in smaller fruit size and hence breeding efforts need to retain fruit size while selecting for decreased canopy. The development of dwarf tomatoes began with the release of Dwarf Champion and more recently with Micro-Tom (Livingston Seed Company, 1896; Scott and Harbaugh, 1989). While Micro-Tom was a concept product, Micro-Tina and Micro-Gemma were released with improved sweetness (Scott et al., 2000). Recently, the performance of dwarf cultivars was tested for plant growth, yield, and CEA fitness (Langenfeld and Bugbee, 2023). Extended greenhouse production times can be compensated by shorter crop cycles and increased planting density in vertical and indoor farms. Plant performance under hybrid or fully electric lighting needs consideration to develop new CEA varieties. CEs also offer opportunities for the implementation of robotics and automation. Therefore, developing traits granting process mechanization such as synchronous maturity and longer pedicels for harvesting ease is essential. Synchronous maturity is a polygenic trait predominantly used in the processing tomato industry for mechanical harvest (Lukyanenko, 1991). *Golden 2-like 2* (*GLK2*) gene has been widely used in modern breeding programs to control uniform fruit ripening. Genes *terminating flower* (*tmf*) and *jointless* (*j*) reduce the number of flowers in the inflorescences of lateral shoots and can be used in maintaining the quality and yield of fruits in dwarf plants in CEs (Molinero-Rosales et al., 1999; MacAlister et al., 2012). Dwarf and early varieties help in spreading the risk over multiple cycles.

While some CEs use sunlight and supplemental electric light, indoor vertical farms solely rely on electric lighting. Focus on genes *eid1* and *lnk2* involved in the circadian clock could help in breeding plants for altered photoperiod to reduce energy costs (Müller et al., 2018). Plants with high phenotypic plasticity can help plants adapt to different light sources and production systems such as hydroponics, aeroponics, or aquaponics. Shoot architecture including leaf number, area, and angle determines light capture, photosynthesis, and transpiration but is highly influenced by the environment. Plant responses due to growing conditions could help develop breeding strategies to capitalize on these traits. Inflorescence branching caused by *FALSIFLORA* and other genes could be used for smaller fruit types like cherry and grape, while *tmf* mutants causing unbranched inflorescences are desirable for Roma and large-fruited tomatoes (Molinero-Rosales et al., 1999; MacAlister et al., 2012).

Besides the traits useful to crop optimization in CEA, several fruit traits pertaining to consumers are significant, such as fruit striping, skin texture, nutritional quality, flavor, shape, size, and aroma. Various studies have inventoried metabolites in tomato fruits as previously reviewed (Rothan et al., 2019). Purple tomatoes with increased anthocyanin content have been developed by combining the dominant allele *Atv* with *Aft* or *Abg* in “Indigo rose” and “Sun Black” cultivars (Ooe et al., 2016; Blando et al., 2019). Genetic engineering has been applied to enhance anthocyanin production using genes encoding R2R3-MYB, bHLH, and WDR factors (Zhang et al., 2019). Overexpression of *S. lycopersicum Anthocyanin 1* (*ANT1)* and *SlAN2* originating from *S. chilense* resulted in enhanced anthocyanin production (Sun et al., 2020). Furthermore, increased anthocyanins using two snapdragon *Delila* (*Del*) and Rosea (*Ros1*) genes delayed overripening and reduced susceptibility to a postharvest disease gray mold (Zhang et al., 2013). The level of anthocyanins produced by genetic engineering using *Del* and *Ros1* is higher than conventionally bred purple tomatoes using *atv* and *Aft* (Baranski et al., 2024). Therefore, both traditional and biotechnological breeding could be applied to increase nutritional compounds (Ilic and Misso, 2012). These resources could be used to develop varieties with novelty in consumer-related traits to minimize market competition and generate price premiums.

A wide range of tomato fruit colors appeal to consumers and some colored pigments are known to prevent cardiovascular disease and potentially reduce obesity (Gammone et al., 2015). Tomato fruit color is a multigenic trait. The red color of tomato is due to all-*trans*-lycopene, naringenin chalcone (NarCh), and yellow skin (Zhu et al., 2018). Mutation in recessive *yellow* (*y*) disrupts NarCh deposition resulting in transparent skin and pink-colored fruits. Loss of function of *PSY1* leads to yellow fruit, and mutations in *CRTISO* and *IDI1* genes in *tangerine* (*t*) and *fruit carotenoid-deficient 1* mutants resulted in overall carotenoid reduction, giving orange color (Isaacson et al., 2002; Pankratov et al., 2016). Accumulation of carotene due to lycopene *β*-cyclase and *ϵ*-cyclase encoded by *CrtL-b* and *CrtL-e* genes also confers an orange color in ripe tomato fruits (Ronen et al., 2000). Brown-colored tomato fruits have been attributed to an inability in chlorophyll degradation due to mutation in *STAY-GREEN1* (*SGR1*) coupled with lycopene accumulation during ripening (Barry et al., 2008). Genetics of carotenoid production is reviewed by Baranski et al. (2024). While *S. lycopersicum* mostly produces red tomatoes, fruits of some *S. cheesmanii* were reported to be yellow, yellow-green, orange, and purple, fruits of *S. pimpinellifolium* were reported to be red, fruits of *S. habrochaites*, *S. peruvianum*, *S. pennellii*, and *S. chmielewskii* were described to stay green, and fruits of *S. neorickii* were reported to be pale green (Peralta et al., 2021). *Crimson* (*og^c^*) gene, an alternative allele at Beta (β) locus, is widely used in fresh market breeding as it increases lycopene and provides deep red color in seed locules and pericarp. In addition to *og^c^*, a dominant QTL (*lyc12.1*) from *S. pimpinellifolium* accession LA2093 increases lycopene by 50% to 70% without reducing β-carotene (Kinkade and Foolad, 2013). Recently, three color-related genes Phytoene Synthase 1 (*PSY1*), R2R3-MYB transcription factor (*MYB12*), and *SGR1* were edited using multiplexed CRISPR-Cas9 to produce yellow, brown, pink, light yellow, pink-brown, yellow-green, and light green–green colors of fruits (Yang et al., 2022).

Tomato flavor is a fusion of sugars, acids, and numerous volatile organic compounds (VOCs). Selection for increased yield, fruit size, and disease resistance has impacted unintentional flavor loss in modern varieties (Folta and Klee, 2016). A breeding opportunity exists to revisit the lost flavor genes and leverage CEA produce for high-quality products (Figàs et al., 2015). The sources and genetics of tomato flavor have been previously studied (Pereira et al., 2021). Although not trivial, improvement in flavor could be achieved through manipulating VOCs without significantly impacting quality or yield with the help of automated phenotyping, SNP markers, and gene editing. While a clear understanding of flavor has not been established, several studies have discovered genomic regions and variants involved in flavor (Kuhalskaya et al., 2024). Genomic regions including genetic loci, candidate genes, and transcription factors (TFs) involved in flavor in tomato have also been reviewed (Kaur et al., 2023). These regions can be harnessed to develop specific flavors in novel varieties for CEA.

### 4.4 Pepper

Pepper (*Capsicum annuum* L.) breeding for CEA mainly emphasizes plant growth and architecture, adaptability to environmental factors (temperature, humidity, and light efficiency), enhanced fruit quality, tolerance to biotic stresses, yield and productivity, WUE, amenable pollination and fruit set, and phenotypic plasticity, among others (Hummer, 2021; Goldstein and Ehrenreich, 2021). Breeding peppers (sweet, hot, and specialty type) for CEA requires an integrated approach to incorporate the targeted traits tailored for specific growing conditions. Breeding for targeted traits involves conventional breeding methods as well as advanced biotechnology approaches including MAS and genome editing. The genetic control of plant architecture is important in CEA breeding and candidate genes like MADS-box protein and WUSCHEL-like genes (*Capanan11g001832* and *Capana00g000667*) are linked to inflorescence architecture in pepper (Lv et al., 2019). A single-base mutation in the *CaBRI1* results in a dwarf phenotype (Yang et al., 2020). The *FASCICULATE* (*FA*) gene primarily known for its effects on fruit clustering also influences plant architecture and branching patterns (Elitzur et al., 2009). These findings offer insights into plant architecture and trait variation for breeding for CEA.

Enhancement in pepper fruit quality attributes like vitamin and antioxidant content as well as uniform fruit size and shape may generate added value at the retail level (Rouphael et al., 2018). Several genes and markers have been identified to improve fruit quality. The Capsanthin-capsorubin synthase (CCS) gene is a critical component in carotenoid biosynthesis, particularly in the production of red pigments (Lefebvre et al., 1998). This gene is closely associated with the dominant *y^+^* allele, which results in red fruit coloration while its absence or mutation leads to the recessive *y* allele resulting in yellow or orange fruits (Popovsky and Paran, 2000). Gene *Capana01g004285*, encoding the BREVIS RADIX (BRX) protein, was linked to the locule number that influences fruit quality (Ma et al., 2022a). Additionally, *Capana10g002229* was proposed to encode a polygalacturonase as a strong candidate gene associated with the deciduous character of ripe fruit impacting fruit softening and abscission (Hu et al., 2023). Furthermore, GS was explored for predicting fruit length, shape, width, weight, and pericarp thickness in pepper highlighting the potential of Reproducing Kernel Hilbert Space (RKHS) as a method with high prediction accuracies (Hong et al., 2020).

Damping off, PM, and bacterial spot are major disease concerns while aphids, whiteflies, and spider mites are the main pest issues observed in pepper production (Messelink et al., 2020). Wild relatives of pepper are valuable sources of resistant genes (Devi et al., 2021). Additionally, species in *Capsicum* have mechanisms such as osmoprotectant production, autophagy, and involvement of TFs and plasma membrane proteins in stress tolerance with identified genes and QTLs contributing to biotic stress tolerance (Jaiswal et al., 2019; Parisi et al., 2020). Numerous studies have pinpointed genomic regions associated with *Phytophthora* resistance (Siddique et al., 2019; Kumar et al., 2022; Ro et al., 2022; Kaur et al., 2024). The utilization of these genetic resources and markers is crucial in developing resilient and high-yielding varieties.

Early and continuous fruit sets can increase pepper productivity in CEA (Gautam et al., 2024). Multi-locus models in diverse populations have revealed eight GBS-derived SNP markers linked to multiple traits, indicating shared genetic control between plant height, width, and yield components (Lozada et al., 2021). QTL analyses have shown high heritability and identified 24 QTLs related to physiological traits influencing yield with pleiotropic effects observed on specific linkage groups (Alimi et al., 2013).

Breeding pepper for CEA could benefit from improving stress resilience and WUE. In bell peppers, two linked QTLs on chromosome 10 control post-harvest fruit water loss (PWL), a trait closely related to WUE (Popovsky-Sarid et al., 2017). These efforts advance the identification of the underlying genes increasing selection efficiency to develop water-efficient and drought-tolerant varieties (Lee et al., 2018).

Solanaceous vegetables (tomato, pepper, and eggplant) often require external pollinators to enhance fruit set and yield; therefore, selection of desirable flower types is necessary to facilitate improved pollination (Schubert, 2017; Folta, 2019). *C. annuum* and *C. chinense* produce single and multiple flowers per node, respectively, and have been the focus of genetic investigations. A study on recombinant inbred lines (RILs) between *C*. *annuum* and *C*. *chinense* identified four QTLs on chromosomes 1, 2, 7, and 11 accounting for 65% of the phenotypic variation in multiple-flower-per-node trait with five candidate genes involved in shoot and flower meristem development (Kim et al., 2022). Transcriptome analysis of different developmental stages of flowers identified several differentially expressed genes (DEGs) involved in flower development, nectar biosynthesis, and nectary development (Deng et al., 2020). The first flower node (FFN) trait important for evaluating fruit earliness was studied using bulked segregant analysis (BSA) and specific-locus amplified fragment sequencing identifying 393 high-quality SNP markers and 10 candidate regions on chromosome 12 associated with FFN (Zhang et al., 2018b). Further QTL mapping identified two major QTLs, Ffn2.1 and Ffn2.2 located in linkage group 2, associated with FFN consisting of 59 candidate genes including three DEGs (Zhang et al., 2019). Six QTLs were identified in an interspecific population developed from *C*. *chinense* and *C*. *annuum* controlling flower number per node. A candidate gene, *Capana02g000700*, encoding the homeotic protein APETALA2, significantly associated with flowering time (Zhu et al., 2019). These studies collectively reveal genetic mechanisms controlling flower type and can be used in breeding programs to improve pepper yield.

Phenotypic plasticity plays an important role in regulating plant growth and development functions that are influenced by the growing environment; hence, breeders must include this trait in their CEA breeding strategy. Phenotypic plasticity in pepper is influenced by various genetic markers and environmental factors. Variability in morphological and biochemical traits observed in *C. baccatum* accessions can enhance fruit length, diameter, fresh mass, and antioxidant activity (Constantino et al., 2020). In wild *C. annuum* populations, genetic differences leading to adaptive phenotypic plasticity based on water and light availability have been observed. Phenotypic plasticity is essential for plant fitness and is influenced by natural selection and genetic drift with selection gradients varying based on resource availability (Romero-Higareda et al., 2022). The genetic basis of phenotypic traits in *C. annuum* has been further elucidated through QTL identification associated with domestication and agronomic traits. These QTLs highlight the genetic architecture underlying traits such as fruit form, seedlessness, and growth habit, providing insights into domestication and exploiting wild alleles for crop improvement (Lopez-Moreno et al., 2023). Additionally, the marker effect networks have been proposed as a novel method to identify genetic markers associated with environmental adaptability. This approach was demonstrated in maize and can be adapted in pepper to understand how different markers co-vary across environments providing insights into phenotypic plasticity and environmental modulation of the genome (Coletta et al., 2023). Together, these studies underscore the complex interplay between genetic markers, environmental factors, and phenotypic plasticity in pepper, enabling breeding and conservation efforts. By exploiting these genetic resources and molecular breeding techniques, future research can focus on optimizing plant architecture, enhancing yield, and improving overall adaptability in CEs. This targeted breeding approach can potentially revolutionize pepper cultivation in CEA, leading to more efficient and productive systems that meet the growing demand for sustainable and high-density crop production.

### 4.5 Cucurbits: cucumber, squash, and melon

Cucurbits such as cucumber (*Cucumis sativus*), melons (*Cucumis melo*), squash (*Cucurbita* spp.), and pumpkins are produced in fields, semi-controlled environments, and increasingly in CEs. For fruiting crops, a long harvesting season is highly desirable for CEA production. Most cucurbits display indeterminate vegetative growth when fruits are continuously removed (Loy, 2004). When not removed, fruits become dominant sinks for photosynthate, and this is accompanied by a slowing or cessation of vegetative growth (Valantin-Morison et al., 2006). Thus, crops with fruits harvested at an immature state like cucumber and summer squash will likely yield more in a CEA system than crops like pumpkin and melon that require full reproductive maturity. For CEA facilities to produce such fruits, niche markets will have to be developed that demand superior fruit quality as they will need to command a high price. Accompanying the increased quality, marketing efforts will be needed that allow for CEA-produced cucurbits to be differentiated in the marketplace via branding and labeling.

Cucurbits face a wide range of pest and disease pressures that can be avoided by growing them in CEs. Insect pests like the squash bug (*Anasa tristis*), squash vine borer (*Melittia cucurbitae*), and striped and spotted cucumber beetles (*Acalymma* spp.) can be excluded from cucurbits by using insect screening (Ingwell and Kaplan, 2019). This reduces both direct damage by insect pests and diseases that they transmit.

Among Cucurbitaceae, cucumber occupies the greatest CEA acreage currently (Pal, 2020). Published genomes and molecular markers are available for the major cucurbits to assist and accelerate breeding for CEA. Cucumber has a small genome with only seven chromosomes (Sun et al., 2006). Not only is it the most widely produced cucurbit in CEA, but also the crop with the most annotated genes. The cucumber genome was first sequenced in 2009 by Huang et al. Currently, three sequenced genomes have been published representing three of the major cultivar groups of cucumber. Approximately 22 QTLs have been mapped in cucumber including QTLs for important traits for CEA like fruit length, early flowering, parthenocarpy, gynoecy, and compact growth (Dey et al., 2023). Additionally, QTLs for important disease resistance traits have been mapped including for DM (Wang et al., 2016), PM (Nie et al., 2015; Xu et al., 2016), Fusarium wilt (Zhang et al., 2015), gummy stem blight (Liu et al., 2017), and cucumber mosaic virus (CMV) (Shi et al., 2018). Additionally, resistance to abiotic stresses such as low temperatures (Song et al., 2018), high temperatures (Dong et al., 2020), and salt stress also have mapped QTLs (Ahmad et al., 2023). Such extensive mapping and linked molecular markers should enable breeders to rapidly advance on breeding new cultivars especially well-suited for CEA.

Cucumber displays a wide range of flowering habits (Dhall et al., 2023). The development of gynoecious cucumber varieties is a major factor in the success of cucumber as a greenhouse crop. Three different loci are thought to affect sex expression in cucumber: *F* confers female flowers with incomplete dominance, *m* is a recessive allele that confers andromonoecy, and *a* confers androecy. The genotype of gynoecious varieties is thought to be *MMFFA/a.* A second locus has also been uncovered that confers gynoecy and is a recessive allele called *gy.* Because the femaleness conferred by *gy* is thought to be more stable than *F*, it is the gene most commonly found in gynoecious cucumber varieties (Dhall et al., 2023). Additionally, most cucumber cultivars used in greenhouse production are parthenocarpic. The *Pc* gene confers parthenocarpy in cucumber and is thought to be incompletely dominant. Genomics research with this trait has revealed great complexity including epistatisis and multiple chromosomal locations, and appears to be inherited quantitatively (Sun et al., 2006).

Although CEA systems can exclude many important insect pests, breeding cucumbers for disease resistance remains a major priority for breeders. Diseases like DM, PM, bacterial wilt, Fusarium wilt, CMV, and watermelon mosaic virus (WMV) can all present challenges for growers in CEs (Singh et al., 2017). Extensive molecular genetics work has revealed QTLs for resistance and breeders use MAS to guide their efforts in breeding resistance to these diseases.

Currently, most cucumber production in CEA systems takes place in greenhouses and high tunnels; however, cucumber is an emerging crop for vertical farms or PFALs (plant factories with artificial lights). Bush cultivars with greatly reduced internode length are available in cucumber and are the most suitable cultivars for vertical farms. Classical genetics studies identified seven genes that affect cucumber plant height and growth patterns, giving breeders the ability to tailor growth habits to a wide variety of growing environments (Naegele and Wehner, 2016). A recessive gene thought to impart a compact or dwarf habit and an associated molecular marker were identified and mapped by Li et al. (2011). This finding should guide breeders to develop compact cucumber cultivars (Li et al., 2011). Other important traits in cucumber breeding include freedom from bitterness, lack of spines on fruits, heterosis when making hybrids, flesh thickness, and yield (Dey et al., 2023).

Most modern cultivars of summer squash and zucchini have a bushy appearance with short internodes and a thickened central stem. Only one bush gene has been named, *Bu*, and is incompletely dominant to vine habit (*bu*). The recessive allele confers a vining habit with long internodes (Loy, 2013). However, the genetics behind the bush trait are likely not as simple as a single gene. Three QTLs associated with the bush phenotype in *Cucurbita pepo* were identified (Xiang et al., 2018) and QTLs for the bush trait had been previously mapped in *C. maxima* (Zhang et al., 2015). Lateral branching is also an important trait for breeding squash for CEA (Loy, 2004). Freedom from lateral branching is desirable as it promotes airflow and light penetration into the plant canopy, allowing for easy access to fruits for harvest. Greenhouse cultivars of summer squash should be single-stemmed with a semi-bush habit that enables trellising and utilization of the vertical space in modern greenhouses. The glabrous trait, conditioned by a single recessive gene (*gl-2*), is also highly desirable for CEA summer squash cultivars. This gene reduces scratching of fruits during the post-harvest period and eases skin irritation for CEA workers caused by trichomes found along stems and leaves of non-glabrous summer squash cultivars (Xiao and Loy, 2007).

A wide range of fruit shapes are available in summer squash including straightneck or marrow, crookneck, discoid or scallop, and round (Paris, 1996). Preferences for fruit shape in summer squash vary greatly. A wide range of colors is also available and range from dark green to light green/gray, yellow, bicolor, and white. Color expression is affected both by genes that impact rind color and by flesh color. Striping, conferred by the *L1/L2* gene complex, is also a feature of some cultivars of zucchini and yellow straight and crookneck squash. The *B* gene causes precocious yellow pigmentation and is found in some varieties of yellow summer squash and yellow or golden zucchini (Shifriss, 1965, 1996). When heterozygous, the *B* gene is known for generating bicolor fruit as in the cultivar “Zephyr”, which is both bicolor and has the *L1* allele for broad normal stripes. When homozygous, the *B* gene turns *Cucurbita* fruits uniformly yellow before anthesis. In addition to the use of *Cucurbita pepo* as summer squash, globally, other species may be harvested immature and eaten as summer squash as in the case of Korean summer squash cultivars of *Cucurbita moschata*, Italian *Cucurbita moschata* cultivars (cv. Trombocino) (Andres, 2004), and bush cultivars of *Cucurbita maxima* in South America (cv. Zapallito de tronco) (López-Anido et al., 2003). Interspecific hybrids of *C. maxima* × *C. moschata* are used as rootstocks for grafting crops like melon (Yarsi et al., 2012), used as winter squash (cv. Tetsakuboto) (Queiroga et al., 2017), and may have use as summer squash. Parthenocarpy in summer squash is a highly desirable trait for the development of cultivars suitable for CEA production. Several parthenocarpic cultivars (cv’s Whitaker, Parthenon, and Golden Glory) are in the marketplace, and breeding efforts are underway globally for the generation of new cultivars (Martínez et al., 2014; Tian et al., 2023). Although molecular breeding in summer squash is not as developed as in other cucurbits, its genome has been sequenced and is available to molecular breeders (Xanthopoulou et al., 2019). Major QTLs for traits such as growth habit, early flowering, leaf morphology, fruit size, and flesh color have already been molecularly mapped (Montero-Pau et al., 2016).

Fungal diseases like PM, caused by *Podoshpaera xanthii* and *Golovinomyces orontii*, pose a significant challenge for summer squash growers in CEA systems (Lebeda et al., 2024). Recently, Lebeda et al. (2024) reviewed the PM of cucurbits. Currently, only one resistant locus, a single, incompletely dominant gene called *Pm-0*, has been incorporated into commercial cultivars of summer squash. Molecular markers for this locus are available commercially for summer squash breeders (Holdsworth et al., 2016). Other resistant loci have been described in other *Cucurbita* species, like *Cucurbita moschata*, but are not yet available in commercial cultivars (Park et al., 2020; Alavilli et al., 2022). Other challenges for summer squash production in CEA include DM (Lebeda and Cohen, 2011) and *Choaenophora* fruit rots (Emmanuel et al., 2021).

CEA production of melons (*C. melo*), primarily cantaloupes also called muskmelons or rockmelons, continues to increase globally (Cantliffe and Vansickle, 2003). Like cucumber, melon plants display a wide variety of flowering habits and the availability of gynoecious lines and male sterile lines assist in its breeding (Hanafi, 2014; Kesh and Kaushik, 2021). Extensive genomic tools are available to melon breeders and many QTLs have been mapped to the melon genome. Melon breeding is facilitated by commercially available molecular markers for important traits such as Fusarium wilt, PM, WMV, and Zucchini yellow mosaic virus (ZYMV) resistance (Shahwar et al., 2023). Because many modern cultivars were bred for disease resistance and long shelf life, opportunities exist to breed for exceptional eating quality for CEA systems. Crops like cantaloupe vary greatly in quality when grown under field conditions leading to dissatisfaction among consumers (Farcuh et al., 2020). Advantages of CEA for melon production include reduced pest and disease pressure (Ingwell and Kaplan, 2019), reduced fruit cracking (Saltveit, 2016), and a reduction in risk from food-borne pathogens by avoiding melon to soil contact (EFSA Panel, 2014), a significant problem in field-based melon production. Additional traits that would help facilitate CEA melon production include parthenocarpy, a dwarf or compact plant stature, and fruiting along the main stem rather than fruiting along lateral branches as is typical of melon cultivars. In the US, cantaloupe, honeydew, and watermelon are the primary classes of melons available in most grocery stores. Specialty melons like Asian melons, casaba melons, Hami melons, casaba melons, Galia melons, and the horned melon (*Momordica charantia*), are becoming more popular and may offer opportunities for CEA production (Duncan and Ewing, 2015). Watermelon, *Citrullus lanatus*, is predominantly produced in open fields in the US, while countries like South Korea extensively use greenhouses for its production (Park and Cho, 2012).

### 4.6 Strawberry

Strawberry has drawn significant attention to CEA growers in the US as new findings demonstrate doubled yields as compared to field production (NIAB, 2022). Low height profile, high market value, wide demand, and high nutritional content make strawberries a suitable candidate for CEA (Hernández-Martínez et al., 2023). The lack of CE-tailored breeding efforts remains an obstacle to the wide application of CE strawberry farming (Hoffman and Shi, 2020). In some European countries, Canada, and Japan, strawberry greenhouse operations using supplemental lighting are standard and cultivars are selected for these settings. In the US, strawberry breeding for CEA needs to focus on selecting plants that perform well under two lighting systems, hybrid and electric lighting, hydroponic and substrate-based production, single and multi-tiered platforms, and resistance to prevalent diseases and pests.

Robotic and automated harvesting technology is being highly investigated in the CE strawberry industry. Breeding efforts need to be geared towards selecting traits supporting mechanical and automated harvesting. In vertical and greenhouse production, fruits hang from tabletop systems supporting automated harvesting. Improving traits like uniform long trusses, and larger and uniform shapes to generate better-displayed fruits can facilitate automation in harvesting (Diamanti et al., 2011). Cultivars like “Camarosa” and “Florida Elyana” are susceptible to yield loss due to misshapen fruits (Chandler et al., 2009; Ariza et al., 2012). Misshapen fruits can result from biological factors like unsuccessful pollination, abnormal carpel development, abiotic factors like rain damage, high temperature, and external factors like insect and disease damages (Chandler et al., 2009). Misshapen fruits can hinder proper recognition during mechanical harvest. Shape uniformity is a complex trait influenced by genetic and environmental factors, but improvements are possible through manipulation of the genetic component (Chandler et al., 2009; Whitaker et al., 2020). A QTL on chromosome 2B controlling fruit uniformity identified in a multi-parental mapping population can be harnessed to select against misshapen fruits and improve fruit uniformity (Li et al., 2020a). Selection of plants for evenly distributed carpels with better pollination abilities and heat tolerance can improve fruit quality in CEs. Breeding efforts in pursuit of parthenocarpy could assist in developing high-quality fruits in CEs where pollinations are suboptimal. Increased peduncle length can help the fruits hang below the canopy. However, the genetic architecture of traits like peduncle length and runner production are time-consuming and labor-intensive to quantify in large breeding sets and are not completely understood. Axillary meristems in strawberries can develop into either runners or inflorescence determining the fate of fruit yield. While runnering is highly desirable for nursery, high runner production during fruit production is undesirable as it incurs an increased cost for trimming and photosynthate translocation to runners compromising fruit yield. Identification of genetic control of flowering and runnering in cultivated strawberry is important to the industry and research (Whitaker et al., 2020). Thus, plant selection is critical for the optimized production of fruits and runners. Integrated research of selecting plants with reduced runnering under modified CE growing conditions along with automated removal of runnering can increase fruit yield. Accessions of diploid woodland strawberry *F. vesca* show variation from no to extreme runner production. Recessive mutations in the Runnering (R) locus cause runnerless plants (Brown and Wareing, 1965). A 9-bp deletion in the *FveGA20ox4* is responsible for the runnerless phenotype (Hawkins et al., 2016). Even when *FveGA20ox4* is mutated, a nonsense mutation in the DELLA protein encoded by *FveRGA1* was found responsible for constitutive runnering (Caruana et al., 2018). The genetics of runnering is completely different in *F. anannassa* from *F. vesca* and is controlled by perpetual flowering and runnering (PFRU) (Gaston et al., 2013). However, the causal gene for runnering in *F. anannassa* is not known.

Strawberries are enjoyed by consumers for their flavor and nutritional benefits. While flavor preference is subjective, its components like sugar–acid balance, texture, aroma, and appearance can be improved through genetics and breeding. In addition to sugars and sugar–acid balance, breeding and selecting plants for high VOCs such as γ-D-galactone; 5-hepten-2-one, 6-methyl, and multiple medium-chain fatty acid esters can enhance fruit flavors and quality. Two QTLs on linkage group 6A controlling different esters production can be used to manipulate aroma strawberries (Fan et al., 2021). Breeding for enhanced VOCs has also been the focus of open-field strawberry breeding programs (Chambers et al., 2014). A negative correlation between soluble sugars and yield requires a need for their balance (Cockerton et al., 2021). Higher concentrations of VOCs enriching flavor and sweetness are observed in ripe fruits with shorter post-harvest shelf life. In field breeding, cultivars are bred for firm skin to withstand long-distance transit, reduce fruit damage during handling, and create a barrier for insects and diseases leaving behind cultivars with superior flavor but coupled with low firmness or disease susceptibility (Moya-León et al., 2019; Zacharaki et al., 2024). Breeding new cultivars with high flavors can capitalize on allowing fruits to ripen longer as these facilities are in or near consumers for CEA. A few QTLs have been reported for SSC; however, their presence and stability across breeding germplasms and environments are low (Gezan et al., 2017; Verma et al., 2017b; Natarajan et al., 2020). Similar growing environments across CEA can be leveraged to discover and use the genomic regions controlling SSC in selecting new cultivars. Furanones like furaneol and mesifurane are attributed to the sweet, caramel odor in strawberries (Ulrich et al., 1997). Genes quinone oxidoreductase (*FaQR*) and o-methyl transferase (*FaOMT*) on chromosomes 1C and 7D are evidenced to be involved in the production of furaneol and mesifurane, respectively (Barbey et al., 2021; Raab et al., 2006). Genes alcohol dehydrogenase (*ADH*), *SAAT*, and *FaAAT2* are reported to confer ester production (Wolyn and Jelenkovic, 1990; Aharoni et al., 2004; Cumplido-Laso et al., 2012) The production of methyl anthranilate, providing grape aroma in strawberries, is modulated by two genes anthranilic acid methyl transferase (*FanAAMT*) and anthranilate synthase alpha subunit 1 (*FaASa1*) (Pillet et al., 2017; Barbey et al., 2021; Fan et al., 2022). Similarly, fatty acid desaturase 1 (*FaFAD1*) and three QTLs on LGVII-1 and 6B and 7B have been tagged to control two lactones, γ-decalactone and γ-dodecalactone, which confer peachy flavor (Sánchez-Sevilla et al., 2014; Oh et al., 2021; Rey-Serra et al., 2022). Linalool and nerolidol that provide floral and citrus-like aroma in strawberries were revealed to be modulated by nerolidol synthase 1 (*FaNES1*) located in chromosome 3C (Aharoni et al., 2004; Fan et al., 2022). Regulation of these genes by controlling the aromatic compound syntheses through marker-assisted breeding and precision breeding could help to develop consumer-desired flavors.

One of the most prevalent diseases in strawberry in CEA is PM. Characterization of pathogen races causing the disease in CEA in the US is needed. In field production, PM is caused by *Podosphaera apahanis.* Inheritance of PM resistance using natural infections in fields and greenhouse study revealed low disease rating correlations between two environments when disease pressure is low and high when the disease pressure is high (Nelson et al., 1995). Quantitative analysis revealed that non-additive dominance was more prominent than additive variance caused by two additive genes with one considerable epistatic gene causing susceptibility (Hsu et al., 2011). Four stable QTLs and a few transients have been observed in Hapil cultivar to confer PM resistance that could be further investigated for CE breeding (Cockerton et al., 2018). Additionally, three *FveMLO* and 12 *FaMLO* susceptibility genes have been identified that could serve as candidates for gene editing and improving host resistance to PM in strawberries (Tapia et al., 2021). Gray mold (*Botrytis cinerea*) could become a threat to CEA strawberry production. However, no known source of resistance has been identified and requires further investigation on host resistance (Petrasch et al., 2019).

## 5 Genome-informed breeding

The genome sequences of crops have enriched our understanding of fundamental crop biology and provided new opportunities for crop improvement. As a result, knowledge obtained from high-quality genomes and re-sequencing data of several CE-grown crops such as lettuce, spinach, tomato, strawberries, peppers, and cucumbers (Huang et al., 2009; Kim et al., 2014; Reyes-Chin-Wo et al., 2017; Xu et al., 2017a; Edger et al., 2019; Hosmani et al., 2019) is available to be used in breeding. Significant progress in enhancing genetic gain in major crops has been achieved using genomic resources in the past decades. However, these resources are lagging in crops with less economic importance. The availability of genomic information will help in cataloging genome-wide spatial and temporal gene expressions, linking functionality to uncharacterized genes, discovering and using epigenetic factors, and establishing genome-wide functional and biological data frameworks. Furthermore, RNA splicing variants, non-protein-coding genes, and regulatory sequences underlying complex traits could be elucidated. While the prediction of genetic merit using genome-based data in association with the phenotype is being achieved for additive effects, novel techniques are needed to incorporate heterosis and epigenetic factors in the equation, especially for complex traits for which phenotyping is the main limiting factor.

Genetic markers are a key aiding tool in selecting desired plants in crop breeding and identifying causal genes associated with phenotypes using unbiased genetic mapping approaches including QTL mapping and GWAS. The discovery of millions of SNPs with simultaneous automation of marker genotyping has markedly reduced the cost per marker, mainly SNPs. In CEA-grown crops, SNP arrays are developed and often improved for strawberries (Bassil et al., 2015; Verma et al., 2017a), tomato (Víquez-Zamora et al., 2013), and pepper (Hulse-Kemp et al., 2016). The availability of a vast number of SNP makers has also expedited QTL mapping with better power and resolution in crop genetics. Some of them have been translated into the development of diagnostic markers (complete linkage with target phenotypes) or allele-specific markers like Kompetitive allele-specific PCR (KASP) (He et al., 2014) tagging desired traits controlled by single or few loci for MAS (Collard and Mackill, 2008). Additionally, GS, an extension of MAS introduced in animal breeding, is an efficient breeder’s tool that reduces breeding cycles by estimating the genetic value of genotypes to select individuals as new parents in breeding programs using DNA markers as a mandatory component. Over the years, GS has been effectively used in cereals to improve yield (He et al., 2017), quality (Schmidt et al., 2016), and disease resistance (Herter et al., 2018).

The development of reference genome and sequence-based SNP discovery may expedite trait mapping with better power and resolution in CEA. In particular, experimental populations either from two parents or from multi-parents or an association panel comprising hundreds of genotypes could be genotyped with sequence-based genotyping methods like a reduced representation strategy offered by genotyping-by-sequencing (GBS) and Diversity Arrays Technology (DArT) or whole genome resequencing approach. These sequence data can then be aligned to the genome sequence and called several thousand to millions of SNP markers. Using statistical methods, QTL or GWAS scan finds associations between SNP markers and specific phenotypes for CEA. The reference genome also provides a base to contextualize the associated markers on the physical chromosomes and facilitate candidate gene discovery. Notably, the molecular basis of pleiotropic effects (Ramsay et al., 2011) might not have been understood well without the help of genome sequence. Furthermore, gene sequence knowledge is a precondition for cloning important genes, which is very time-consuming and labor-intensive. This situation has been greatly relieved with the generation of the genome sequence, especially in complex polyploid genomes (Athiyannan et al., 2022; Yu et al., 2022).

Genetic characterization of germplasm and genebank collections becomes a reality with the help of reference genomes and cost-effective genotyping platforms like GBS or DArT. In turn, we may mobilize diversity from a cold room to plant factories. The generation of genome sequencing in different genotypes of a species clearly showed that genomic structural variants (approximately >50 nt) including presence/absence and chromosomal rearrangements like inversions and translocations are also prevalent and play key roles in trait innovations and adaptations in plants (Gabur et al., 2018; Yuan et al., 2021). Thus, capturing sequence variants beyond SNPs is also important for a better understanding of genome-to-phenome relationships. Such variants may not be identified with only a single reference genome and, thus, multiple genomes representing different subpopulations or geography are required. This concern is being addressed in the concept of “Pangenome” referring to the entirety of sequence variations in a crop and its progenitor or collections of genomes from the primary gene pool (Jayakodi et al., 2021). Among CE crops, tomatoes have been studied extensively in the frame of pangenome. In 2019, the first tomato pangenome was built using resequencing data and revealed new genes missing in the first reference genome (Gao et al., 2019). Shortly after, 32 *de novo* tomato genome assemblies were generated to construct the second version of the pangenome (Zhou et al., 2022) that improved the sequence read mapping and allowed to capture missing heritability (Gao et al., 2019). In parallel, the tomato pangenome was further enhanced by long-read sequencing of 100 diverse genomes and demonstrated the role of structural variants in fruit weight and flavor (Alonge et al., 2020). Recently, a super-pangenome, integrating crop wild relatives, was constructed in tomatoes. Moreover, a pangenome for pepper was constructed using five genotypes and cataloged genomic structural variants (Liu et al., 2023). Similarly, a graph-based pangenome was developed for cucumber with 11 genomes and provided a variation map for breeding (Li et al., 2022). In strawberry, the first version used five diploid *Fragaria* species (Qiao et al., 2021) and described the fruit color variations. Explicitly, in a breeding population, the pangenome approach could be employed to generate genome assemblies of a set of founder lines to provide a graph-based haplotype map, called the Practical Haplotype Graph (PHG), representing the diversity in the breeding population. The progeny populations tend to be contained within the set of founder haplotypes and, hence, the PHG could be used to impute the missing sequence information in new progeny genotypes. As a result, this robust framework bestows breeders with accurate genotyping and running GS with multiple sequence-based genotyping platforms. Such PHG can be constructed for breeding germplasm specifically and allow the capturing of diverse genetic variants to facilitate CE crop breeding.

Epigenetic variation could be used as markers in crop breeding to improve crop performance and adaptation (Kakoulidou et al., 2021). Already, the heritable epigenetic variations underpinning important agronomic traits in various crop species have been discussed elsewhere (Gupta and Salgotra, 2022). Notably, the analysis of chromatin stats enabled the identification of functional genes and *cis*-regulatory elements (CREs) including promoters and enhancers. The *cis*-regulatory variants render a new source of allelic diversity for breeding (Rodríguez-Leal et al., 2017). For example, manipulation of *cis*-regulatory alleles in tomato provided quantitative variations for inflorescence traits (Rodríguez-Leal et al., 2017). Very recently, a new promotor editing system was established to efficiently introduce quantitative trait variation in crops (Zhou et al., 2023). However, identifying such *cis*-regulatory sequences is a key component to proceeding forward in this direction. Hence, various sequence-based assays including ATAC-Seq (the assay for transposase-accessible chromatin with high-throughput sequencing), chromatin immunoprecipitation sequencing (ChIP-seq), chromosome conformation sequencing (Hi-C), and methylation sequencing including bisulfite sequencing (Lister et al., 2008) and enzymatic conversion (Feng et al., 2020) methods have been developed for detecting chromatin accessibility, CREs including enhancers and promoters, TF binding sites, and methylation profiles. Analyzing and interpreting these datasets may enable the integration of epigenomic information into CEA breeding strategies. Gene expression changes allow a crop to produce different proteins, leading to differences in cell function. Therefore, the exploration of gene functions in CEA may facilitate the discovery of key genes. Collectively, combinations of these omics resources and analyses may provide a holistic view of phenotypic plasticity and trait expression under CEA. Genomic information can efficiently be translated with modern genome editing tools such as clustered regularly interspaced short palindromic repeats (CRISPR/Cas9) (Zaidi et al., 2020) also referred to as new breeding techniques.

## 6 Precision breeding

Advances in gene editing technologies have emerged as powerful tools for precision breeding in horticultural crops. Technologies such as TALENs (Transcription Activator-Like Effector Nucleases), ZFNs (Zinc Finger Nucleases), and CRISPR/Cas systems enable targeted trait modifications, optimizing crops for CEA (Karkute et al., 2017; Limera et al., 2017). Recent innovations in multiplexed genome editing offer greater precision in genetic modifications, reducing the likelihood of unintended effects (Mishra et al., 2021; Molla & Yang, 2020). Additionally, transgrafting offers an alternative precision breeding strategy by combining genetic engineering with traditional grafting methods (Limera et al., 2017). These technologies allow researchers to make precise changes to plant genomes, enhancing desired traits or removing undesirable ones.

The application of gene editing in horticulture has grown rapidly since the first successful genome editing in *Brassica oleracea* using TALEN (Sun et al., 2013), primarily using CRISPR systems (Xu et al., 2019). This surge in genome-editing studies (**Table 1**) reflects the pressing need to adapt crops to the unique conditions of CEA, addressing both current challenges and anticipated demands of next-generation indoor farms. As the CEA industry evolves, researchers are focusing on genes affecting development, metabolism, and stress responses in various horticultural crops to tailor key traits desired for CE-grown crops to the unique conditions of controlled environments. Several key traits are being tailored to the unique conditions of CEA to improve yield, quality, and resource use efficiency.

**Table 1:** A list of publications on genome editing involved in controlled environment agriculture traits in horticultural crops.

Targeted Gene	Tools	Function or Phenotype	Crop	Reference
Plant Architecture				
*BolC.GA4.a*	CRISPR	GA response and dwarfism	Cabbage	Lawrenson et al., 2015
*PROCERA*	CRISPR	GA response and dwarfism	Tomato	Tomlinson et al., 2019
*PROCERA*	TALEN	GA response and taller plant	Tomato	Lor et al., 2014
*SP5G*	CRISPR	Rapid flowering	Tomato	Soyk et al., 2017; Li et al., 2018a
*SP*	CRISPR	Compact plant architecture	Tomato	Li et al., 2018a
*L1L4*	ZFN	Plant architecture	Tomato	Hilioti et al., 2016
*Solyc12g038510*	CRISPR	Jointless and branching	Tomato	Soyk et al., 2017
*FRIGIDA*	TALEN	Vernalization and flowering	Cabbage	Sun et al., 2013
Photosynthetic Efficiency and Light Utilization				
*SBPase*	CRISPR	Leaf senescence	Tomato	Ding et al., 2016
Nutrient Utilization Efficiency				
*StMYB44*	CRISPR	Phosphorus homeostasis	Potato	Zhou et al., 2018
*FLAs*	CRISPR	Phosphorus stress	Rapeseed	Kirchner et al., 2017
Pest and Disease Resistance				
*SlJAZ2*	CRISPR	Resistance to bacterial speck	Tomato	Ortigosa et al., 2019
*Solyc08g075770*	CRISPR	Fusarium wilt susceptibility	Tomato	Prihatna et al., 2019
*Coat protein, from TYLCV*	CRISPR	Resistant to yellow leaf curl virus	Tomato	Tashkandi et al., 2018
*SlMlo1*	CRISPR	Powdery mildew resistance	Tomato	Nekrasov et al., 2017
*Coilin gene*	CRISPR	Resistant to biotic and abiotic agents	Potato	Khromov et al., 2018; Makhotenko et al., 2019
*WRKY11, WRKY70*	CRISPR	Enhanced biotic resistance	Rapeseed	Sun et al., 2018
*eIF4E*	CRISPR	Enhanced viral resistance	Cucumber	Chandrasekaran et al., 2016
*ALS*	CRISPR	Increased herbicide resistance	Watermelon	Tian et al., 2018
*CaERF28*	CRISPR	Anthracnose resistance	Pepper	Mishra et al., 2021
*eIF4E*	CRISPR	Virus resistance	Melon	Pechar et al., 2022
*SlMYC2*	CRISPR	Resistance to Botrytis cinerea	Tomato	Shu et al., 2020
Yield and Product Quality				
*PL*, *PG2a*, and *TBG4*	CRISPR	Cell wall and firmness	Tomato	Wang et al., 2018b
*AP2a*, *NOR, FRUITFULL*	CRISPR	Fruit development and ripening	Tomato	Wang et al., 2019
*SlEIN2, SlERFE1, SlARF2B, SlGRAS8, SlACS2, SlACS4*	CRISPR	Ethylene response and fruit development	Tomato	Hu et al., 2019
*SlDML2*	CRISPR	DNA methylation and fruit ripening	Tomato	Lang et al., 2017
*RIN*	CRISPR	Ethylene and fruit ripening	Tomato	Ito et al., 2017
*SlORRM4*	CRISPR	RNA editing and fruit ripening	Tomato	Yang et al., 2017
*ALC*	CRISPR	Shelf life	Tomato	Yu et al., 2017
*Solyc12g038510*	CRISPR	Jointless mutant, abscission	Tomato	Roldan et al., 2017
*L1L4*	CRISPR	Fruit metabolism during ripening	Tomato	Gago et al., 2017
*SBE1, StvacINV22*	TALEN	Sugar metabolism	Potato	Ma et al., 2019
*FaGAST1*	CRISPR	Increased fruit size	Strawberry	Abdullah et al., 2021
*CmACO1*	CRISPR	Extends the shelf life	Melon	Nonaka et al., 2023
*FAD2*	CRISPR	Fatty acid metabolism	Rapeseed	Okuzaki et al., 2018
*FAE1*	CRISPR	Reduced long-chain FA	Camelina	Ozseyhan et al., 2018
*CsDGAT1, CsPDAT1*	CRISPR	Altered fatty acid content	Camelina	Aznar-Moreno and Durrett, 2017
*FAD2*	CRISPR	Reduced levels of polyunsaturated fatty acids	Camelina	Jiang et al., 2017; Morineau et al., 2017
Visual, Sensory, and Nutritional Attributes				
*SlMYB12*	CRISPR	Pink tomato fruit color	Tomato	Deng et al., 2018
*FvMYB10, FvCHS*	CRISPR	Anthocyanin biosynthesis	Strawberry	Xing et al., 2018
*DcCCD4*	CRISPR	Different colored in carrots	Carrot	Li et al., 2021
*F3H*	CRISPR	Anthocyanin biosynthesis	Carrot	Klimek-Chodacka et al., 2018
*F3H*	CRISPR	Altered flower pigmentation	Wishbone flower	Nishihara et al., 2018
*InDFR-B*	CRISPR	Anthocyanin biosynthesis	Petunia	Watanabe et al., 2017
*C3H, C4H, 4CL, CCR, IRX*	CRISPR	Lignocellulose biosynthesis	Orchid	Kui et al., 2017
*InCCD4*	CRISPR	Altered petal color	Petunia	Watanabe et al., 2017
*SmCPS1*	CRISPR	Tanshinone biosynthesis	Red sage	Li et al., 2017
*GGP1*	CRISPR	Vitamin C biosynthesis	Tomato	Li et al., 2018a
*Psy1, CrtR-b2*	CRISPR	Carotenoid metabolism	Tomato	D’Ambrosio et al., 2018
*Carotenoid isomerase, Psy1*	CRISPR	Carotenoid metabolism	Tomato	(Dahan-Meir et al., 2018)
*SGR1, Blc, LCY-E, -B1, -B2*	CRISPR	Increased lycopene content	Tomato	Li et al., 2018a
*ANT1*	CRISPR/TALEN	Anthocyanin biosynthesis	Tomato	(Čermák et al., 2015)

Traits like compact plant architecture and rapid growth allow efficient use of vertical space and quicker crop cycles in CEA. CRISPR/Cas9 has been used to modify genes like *Solyc12g038510*, *SELF-PRUNING* (*SP*), *PROCERA*, and gibberellic acid inhibitory gene (*GAI*) in tomato; *BolC.GA4.a* (an ortholog of AtGA4) in cabbage (*Brassica oleracea*); and *CsCYP85A1* in cucumber to achieve compact varieties (Lor et al., 2014; Lawrenson et al., 2015; Soyk et al., 2016; Li et al., 2018b; Wang et al., 2018b; Tomlinson et al., 2019). The *DWARF 1* (*DW1*) and *DWARF14* (*DW14*) genes in *Arabidopsis* have also been identified as potential targets for developing compact plants (Hong et al., 2003; Bennett et al., 2006). These genetic modifications increase plant fitness and allow frequent harvests in CEA thereby maximizing productivity.

Enhanced photosynthetic efficiency and artificial lighting utilization can potentially reduce energy consumption while maintaining or increasing yield, contributing to the economic viability of indoor farming operations. In tomato, a model crop for many CEA applications, editing of genes such as *SBPase* (Sedoheptulose-1,7-bisphosphatase), *SlGLK2*, and *SlPIF4* (Phytochrome Interacting Factor 4) has yielded promising results. These modifications have led to enhanced photosynthetic activity and improved light response mechanisms (Powell et al., 2012; Ding et al., 2016; Rosado et al., 2016). Furthermore, overexpressing *GLK* genes and strategic manipulation of light signaling pathways have also shown significant potential in optimizing the response of plants to light in various crops, suggesting broad applicability in CEA crops (Powell et al., 2012; Wu et al., 2012; Nguyen et al., 2014; Kui et al., 2017; Kreslavski et al., 2020).

Efficient nutrient utilization is fundamental for CEA sustainability, reducing input costs and environmental impacts. Modifications to the *StMYB44* gene in potato have shown promise in enhancing nutrient management efficiency. Similarly, in rapeseed, editing Fascilin-like arabinogalactan protein gene has yielded positive results in nutrient utilization (Kirchner et al., 2017; Zhou et al., 2018). While much of the pioneering work in this field has been conducted in cereal crops, the insights gained from these studies pose significant potential for application in horticultural species. Notable examples include the modifications of *OsNRAMP5* and *OsITPK6*, involved in manganese and iron transport, and phosphorus utilization, respectively, in rice (Tang et al., 2017; Jiang et al., 2019). The successful translation of these findings to horticultural crops could revolutionize nutrient management in CEA systems.

The application of gene editing in developing pest and disease resistance is crucial for reducing pesticide use in CEA. In tomato, editing of the *SlJAZ2* gene improved resistance to tomato yellow leaf curl virus (Ortigosa et al., 2019). For bacterial and fungal resistance, editing *SlDMR6* in tomato conferred broad-spectrum bacterial resistance (de Thomazella et al., 2021). Targeting *Solyc08g075770* in tomato and *CaERF28* in chili pepper enhanced resistance to PM and *Phytophthora capsici*, respectively (Prihatna et al., 2019; Mishra et al., 2021). Moreover, multiplexed gene editing, an emerging trend, can facilitate gene pyramiding promising more robust and resilient crops for CEA.

CRISPR/Cas9 has been used to modify genes involved in various yield and quality-related traits. Editing ripening-related genes and ethylene response in tomato has improved fruit shelf life and quality (Yu et al., 2017; Wang et al., 2019). Recent work targeting the DNA methylation pathway showed delayed ripening and extended shelf life (Lang et al., 2017). Modification of steroidal glycoalkaloid metabolism in potato has enhanced tuber quality (Nakayasu et al., 2018). In tomato, editing of *SlGAD2* and *SlGAD3* genes increased γ-aminobutyric acid (GABA) content, enhancing both nutritional value and stress tolerance (Li et al., 2018a). Gene editing has been used to increase fruit size in strawberry (Abdullah et al., 2021) and tomato (Rodríguez-Leal et al., 2017). In cucumber, editing of *CsWIP1* improved fruit yield and quality (Hu et al., 2019).

Precision breeding has revolutionized the enhancement of visual, sensory, and nutritional attributes in CEA-grown crops. Editing genes like *SlMYB12* in tomato and *DcCCD4* in carrot have modified fruit and root color (Klimek-Chodacka et al., 2018; Li et al., 2021). In lettuce, *LsBBX11* editing altered anthocyanin accumulation, affecting leaf color (Park et al., 2019). Modification of genes like *CrtR-b2* in tomato has altered fruit flavor (D’Ambrosio et al., 2018). In strawberry, FanF3H editing improved fruit flavor and aroma (Zhou et al., 2020). Nutritional enhancements include increased β-carotene in banana by targeting *LCYϵ* (Kaur et al., 2020), reduced anthocyanin in strawberry by editing *MYB10* and *CHS* (Xing et al., 2018), and higher vitamin C content in lettuce by targeting *LsGGP2* (Zhang et al., 2018a). Recent work in tomato has also increased lycopene content through *SlMYB72* editing (Li et al., 2020b).

Current gene editing technologies often create insertions or deletions (indels) that result in loss-of-function mutations; future research should focus on gain of function by identifying and manipulating negative regulators of traits critical to CEA. Given the artificial lighting, targeting genes involved in photoreceptor signaling, circadian rhythms, and shade avoidance responses could lead to better adaptation to electric lighting. Genes controlling gravitropism, stem elongation, and leaf angle could be targeted to develop crops better suited for vertical farming systems. Identifying and modifying genes involved in thermotolerance could contribute to energy saving. Enhancing the nutritional content of CEA crops could add value and address specific dietary needs.

## 7 High-throughput phenotyping

Cutting-edge technologies in plant phenotyping have made significant progress in the ability to assess complex traits and physiological factors underpinning crop performances with high precision. Phenomics rely on imaging techniques able to detect the wavelengths generated by the interaction of plants with the electromagnetic light spectrum by measuring the percentage of energy reflected, absorbed, and transmitted in visible and short-wave infrared regions (Williams et al., 2018). The importance of high-throughput phenotyping (HTP) lies in its ability to non-destructively provide valuable insights into plant responses to changing environmental conditions. Plant shape and architecture can be depicted by using sensors detecting the light absorbed by leaf pigments in the visible range and infrared regions, by detecting multispectral and thermal signatures (Tripodi et al., 2022). Sensors allow inference of canopy structure, physiology, and health status of crops response to different growing conditions. Therefore, the advantage relies on dissecting any specific stresses and on quantifying the effect of environmental factors (Tripodi et al., 2018). Implementing automation for plant trait assessment enables researchers to identify stress-responsive genes and improve crop resilience and productivity. It also aids in the development of new crop varieties through MAS and QTL identification, leading to more efficient and targeted plant breeding (Crain et al., 2018). Another key advance of robotics for HTP is the capability to non-destructively assess plant traits efficiently monitoring the growth cycle over time by acquiring data continuously (Fahlgren et al., 2015). HTP in CEA allows a setup of the experiment guaranteeing its reproducibility and better controlling the effect of genotype and environment. However, drawbacks may occur because of the narrowed spectrum of environmental parameters to measure (Fahlgren et al., 2015).

Significant gains in precision and the ability to trace the growth process are made possible by indoor non-invasive phenotyping (e.g., root morphology and recording of diurnal transpiration profiles within days and across the lifetime). Accurate dissection of phenotypic characteristics and performance of plants is therefore possible, providing essential information for the development of resilient crop varieties that can thrive in the face of climate change and other environmental challenges (Langstroff et al., 2021). Dynamic and static approaches can be adopted to achieve accuracy in the assessment.

The main challenge of automated phenotyping is given by the massive amount of data generated by imaging and remote-sensing platforms requiring strategies that guarantee their archiving, access, and analysis. Artificial intelligence (AI) has the ability of sensing devices to automatically carry out analytical and data interpretation tasks through data mining and machine learning (ML). These approaches address the needs for robotics in agriculture (Bini et al., 2020). ML comprises modeling techniques capable of recognizing patterns in a dataset used for decision-making. However, the application of ML requires high-quality data for model training and appropriate software and hardware configuration for the extraction and analysis of elements from imaging, requiring supervision (Janiesch et al., 2021). In recent years, deep learning (DL) techniques have provided remarkable advancements in pre-processing techniques and learning algorithms with training models flexibly handling non-structured datasets (Janiesch et al., 2021; Tripodi et al., 2022). These approaches can help in increasing prediction and selection accuracy in CEA breeding.

## 8 Prospects—tree-bearing fruits and brambles

CEA production is predominantly limited to annual crops due to their small size, fast growth, and rapid harvest intervals. However, many high-value fruit and nut crops are woody perennials that are temperate such as cane fruits (blackberry, red raspberry, and black raspberry), vines (grapes, kiwi, and hops), brambles (blueberry), and tree fruits (stone fruit, pome fruit, citrus, pecan, etc.). Consequently, these plant species are generally not regarded as potential CEA crops due to their large size, requirements for dormancy, and seasonal production. The increasing threats of invasive pests, diseases, and climate change have placed even more pressure on perennial crop growers, prompting them to examine new production systems. Consequently, there has been a shift toward methods that reduce juvenility periods and increase planting densities via changes in horticultural and production practices. These have centered on the use of available genetics such as specialized rootstocks to reduce size and juvenility as well as protected production systems to minimize impacts of pests/diseases and adverse climate events. These changes highlight the importance of investing in future CEA production systems that have the potential to mitigate crop losses, minimize pesticide and chemical inputs, reduce water use, and increase fruit quality by eliminating the need for premature harvest and long-distance shipping, and providing year-round products to consumers.

The transformative potential of rootstocks to perennial crop production is best exemplified in apple. The availability of dwarfing and precocious rootstocks has globally shifted apple production from free-standing trees planted at low density (300–400 trees per acre) to high-density systems that sometimes exceed 3,000 trees per acre that can be grown as fruiting walls to enable automation (Campbell et al., 2023). The severe dwarfing capacity of apple rootstock leads to very slender trees that flower within 1–2 years of planting. Such trees can be placed as close as 18–24" inches apart on a trellis system and pruned to maintain a short planar production platform. While such orchards are significantly more expensive to create, they produce a faster return on investment, increase annual yields per acre, result in higher quality fruit due to better sun exposure, and are more effective in terms of pesticide applications. Although such rootstocks are not currently available for most other crops, it is likely that similar genetics may be available in the crop germplasm and numerous ongoing efforts are underway to identify and exploit them. Rootstocks have been shown to not only alter plant size and juvenility, but also induce changes to scion dormancy, chill requirement, bloom time, and growth habit (Fazio, 2021). Mobilizing these rootstock traits via breeding and/or biotechnology could result in specific crop adaptations that could overcome some of the critical impediments to CEA production.

Protected growing systems for perennial crops are becoming increasingly important for a variety of reasons. In cane berries, high tunnel production has become standard in California and Europe. Covered production minimizes impacts of pests/diseases, enables harvest while raining, and extends the production season. For primocane fruiting varieties (where the flowers produced are the current year’s growth), protected production has been shown to increase yields and minimize pesticide requirements as well as mitigate losses to weather events (Demchak, 2009; Rom et al., 2010; Yao, 2018; Cormier et al., 2020). In sweet cherry, protected production in high tunnels or greenhouses is growing in popularity around the world due to the high market value of fresh cherries and their significant production challenges. While investment costs are high, abiotic stresses are becoming increasingly problematic including unseasonable climate conditions such as frost or excessive rain during fruit development resulting in fruit cracking. Protected cherry growing systems rely on dwarfing rootstocks to maintain small tree size or by growing trees in smaller pots. Likewise, it has been shown that controlled environment production can increase cherry yields and result in larger fruit (Meland et al., 2019). In the case of citrus, HLB disease is threatening US and global production. The lack of genetic resistance along with increased abiotic stress factors and regional economic conditions has reduced citrus production in Florida to less than 10% of what it once was. Meanwhile, HLB has spread across Texas and continues to threaten the California industry. In Florida, some growers have begun developing protected production systems to prevent infection by the psyllid vector (Schumann et al., 2022). While expensive, such systems may be a requirement in the future to maintain US citrus production, and at the same time, they highlight the potential importance of CEA as a future alternative to conventional growing systems in such events where they become unsustainable.

In addition to fruit production, there is a growing role for CEA in nursery stock production. Currently, woody perennials are propagated through a variety of methods including rooting of cuttings, stool beds, grafting, and more recently via tissue culture. A significant challenge for nursery industries is producing high-quality plant materials that are free from pests or diseases. This is typically done under a protected growing system, a greenhouse, or, in the case of tissue culture, a CEA facility. CEA systems such as indoor farms could solve several key challenges. Many pathogens are spread by insect vectors that are incredibly difficult to control such as aphids, psyllids, thrips, or other pests that pose a persistent challenge to nursery propagation. Even if propagated in tissue culture, plant materials of sufficient size need to be grown out in a greenhouse or field prior to distribution. Second, CEA systems have the potential to improve woody plant quality and reduce juvenility through manipulation of temperature, day length, nutrition, and CO_2_ levels—becoming an enabling technology for high-density fruit growers to realize even faster returns on investment, particularly for crops in which precocity is not readily achieved through rootstocks or other horticultural practices. By automating production methods, CEA systems have the potential to produce elite, pathogen-free nursery stock for conventional growers much faster than current industry practices are capable of.

There are many opportunities to develop woody perennial germplasm specifically adapted for CEA systems. Mutants with altered dormancy, flowering time, juvenility, parthenocarpy, and growth habit (such as dwarfing) are abundant in perennial germplasm. Such traits are often incompatible with conventional field production and thus have not been used except for home gardens or as ornamentals. Traditionally, breeding these traits into perennial woody crops takes many decades or even centuries due to the long juvenility period and the need to incorporate numerous traits into a single variety such as growth/flowering habit, self-compatibility, dormancy and chill requirement, abiotic stress resilience, disease and pest resistance along with numerous pomological traits including fruit size, color, shape, pedicel properties for harvestability, firmness, flavor, ripening characteristics, and shelf life. However, it is safe to assume that breeding programs for CEA would be significantly faster. First, the requirements for abiotic stress resilience and disease resistance are significantly reduced. Second, separate breeding programs for rootstock and scion varieties may be unnecessary since the advantages of the root system may not be realized in soilless growth conditions. Likewise, the need for premature fruit harvest to enable long-term storage and packing for long-distance shipping is reduced. Lastly, CEA systems themselves enable the shortening of juvenility period and ensure more rapid breeding cycles with the absence of impacts from plant pests, diseases, and adverse weather events, not to mention consistent growth conditions that minimize variability in fruit quality.

In addition to conventional breeding, biotechnology offers tremendous opportunities to adapt woody perennial crops especially for CEA systems. Using either transgenic or gene-editing approaches, it is possible to alter traits that are otherwise genetically fixed in each species. Overexpression or mutation of key flowering regulators in plum, apple, citrus, grape, pear, and blueberry has shown to lead to temperate tree crops that lack dormancy requirements and flower continually (Endo et al., 2005; Flachowsky et al., 2011; Srinivasan et al., 2012; Song et al., 2019; Tomes et al., 2023). Plant size and shape can likewise be readily manipulated by altering genes associated with hormone responses or gravitropism to generate plants suited for a broad range of production systems (Waite and Dardick, 2021). The potential to shift carbohydrate partitioning into woody crops from wood (i.e., cellulose, hemicellulose, lignan, and lignin) to fruit in CEA production systems where significant structural support is not needed could, in theory, lead to improvements in productivity and fruit quality. Collectively, these technologies may allow us to re-imagine how many of our perennial crops are produced to achieve high-quality, year-round availability from CEA.

## 9 Conclusion

The future of CEA lies in the synergy between cutting-edge biological innovations and smart farming technologies. Focusing on the discussed trait targets and emphasizing fundamental research to identify key regulatory genes can pave the way to CEA breeding. This approach has the potential to significantly contribute to improving the economic viability of CEA operations and ultimately global food security, nutritional quality, and sustainable urban development. As CEA continues to evolve, the integration of plant breeding with advanced environmental control systems and AI-driven management practices promises to revolutionize urban agriculture. However, as these technologies progress, it will be crucial to address regulatory challenges, ensure public acceptance, and continue refining editing techniques to minimize off-target effects. The impact of CE production stands on three pillars of sustainability—economy, environment, and society—and could emerge as the “go to” farming system as open-field production grapples with a lack of agricultural lands, limited input supplies, and the effects of climate change. One challenge facing breeders for CEA is the high operating costs of maintaining sufficiently large areas for growing and selecting within large populations of segregating plants. Obtaining desirable phenotypes with multiple traits often requires growing large populations of plants. Fortunately, tools like MAS, CRISPR, and GS can greatly reduce the number of plants required by culling undesirable genotypes before their planting.

The breeding community aims to leverage the genome and phenome technologies to (1) develop species-specific genomic resources including well-annotated reference assemblies, and assessment of genetic diversity; (2) generate larger marker sets to conduct genome-enabled parentage and traceability; (3) develop HTP and automated phenotyping platforms to collect phenotypic and environmental data and integrate with genomic information; (4) develop models and algorithms for precise management of CE crop production such as nutrient management and lighting responses; and (5) apply genome-informed breeding with the application of gene editing to overexpress or mutate causal alleles to optimize genetic improvement. Most plant germplasms have been primarily developed and selected for open-field production. Therefore, the selection of plant materials based on phenomic and genomic information will increase breeding efficiency in building germplasm and cultivar development for CEA. While genome-informed breeding can facilitate the selection of germplasm for CEs, existing germplasm, especially wild relatives, can be used to harness traits, such as PM and white fly resistance, and heat and drought stress.

The application of omics approaches enables CEA breeding to respond to future problems and address emerging opportunities like plant-based production of nutraceutical compounds in CEs. Regulations on public release and consumption of genetically transformed and gene-edited crops for CEA production will also impact the industry, which needs to be addressed as new materials become available for CEA production. The USDA allows the food produced in CEs under hydroponic, aquaponic, and aeroponic systems to be labeled as organic through certification (USDA, 2018). While Europe does not consider hydroponics as an organic system yet, certified organic soilless production opens a new breeding avenue for CEs in the US. Breeding crops for CEA represents a strategic approach to optimizing production, addressing various challenges and opportunities unique to these environments.

As the CEA industry progresses, next-generation indoor farms are likely to focus on cultivating higher-value crops such as fruiting vegetables, nursery propagules, and plants producing nutraceuticals, medicines, and pharmaceuticals. While CEA systems for perennial woody fruits are not likely to emerge in the immediate future, the adoption of higher-density perennial crop-growing methods along with protected production systems highlights that some of these industries are already moving in that direction. Perennials that have a small harvest window and are fragile with a short shelf life could see significant investment in breeding and technologies for adoption. Integration of these high-value crops in CEA will require advanced precision breeding techniques to ensure trait optimization, crop viability, and marketability. Ultimately, consumers would benefit from having year-round access to normally seasonal fruits that are high quality, locally sourced, nutritious, and affordable. These advancements enable the development of crop varieties tailored to the unique conditions of CEA, contributing to more sustainable, efficient, and profitable production systems. Integrating these techniques with ongoing advancements will be crucial for developing high-quality, nutritious horticultural crops and enhancing global food security and sustainability.
